# Community-Based Child Food Interventions/Supplements for the Prevention of Wasting in Children Up to 5 Years at Risk of Wasting and Nutritional Oedema: A Systematic Review and Meta-Analysis

**DOI:** 10.1093/nutrit/nuaf041

**Published:** 2025-04-24

**Authors:** Zohra S Lassi, Zahra A Padhani, Anna Ali, Komal A Rahim, Maha Azhar, Hamna Amir Naseem, Rehana A Salam, Jai K Das, Zulfiqar A Bhutta

**Affiliations:** School of Public Health, Faculty of Health and Medical Sciences, The University of Adelaide, Adelaide, SA 5000, Australia; Robinson Research Institute, Adelaide Medical School, Faculty of Health and Medical Sciences, The University of Adelaide, Adelaide, SA 5006, Australia; School of Public Health, Faculty of Health and Medical Sciences, The University of Adelaide, Adelaide, SA 5000, Australia; Robinson Research Institute, Adelaide Medical School, Faculty of Health and Medical Sciences, The University of Adelaide, Adelaide, SA 5006, Australia; Robinson Research Institute, Adelaide Medical School, Faculty of Health and Medical Sciences, The University of Adelaide, Adelaide, SA 5006, Australia; Centre of Excellence in Trauma and Emergencies (CETE), Aga Khan University Hospital, Karachi 74800, Pakistan; Dean’s Office, Medical College, Aga Khan University Hospital, Karachi 74800, Pakistan; Institute for Global Health and Development, Aga Khan University, Karachi 74800, Pakistan; Institute for Global Health and Development, Aga Khan University, Karachi 74800, Pakistan; The Daffodil Centre, The University of Sydney, a joint venture with Cancer Council NSW, Sydney, NSW 2011, Australia; Institute for Global Health and Development, Aga Khan University, Karachi 74800, Pakistan; Department of Paediatrics and Child Health, Division of Woman and Child Health, Medical College, Aga Khan University, Karachi 74800, Pakistan; Institute for Global Health and Development, Aga Khan University, Karachi 74800, Pakistan; Centre for Global Child Health, Hospital for Sick Children, Toronto, ON M5G 1X3, Canada

**Keywords:** wasting prevention, under 5 children, community

## Abstract

**Context:**

Malnutrition poses a significant threat to child health, with millions of children worldwide affected by wasting, which increases the risk of morbidity and mortality.

**Objective:**

In this study we sought to evaluate the effectiveness of community-based infant/child food interventions and supplements for preventing wasting among children up to 5 years at risk of wasting and nutritional oedema. The World Health Organization commissioned this review to update their guidelines on wasting due to malnutrition in children.

**Data sources:**

Nine databases were searched from inception until July 2021 and an updated search was carried out on MEDLINE and Ovid MEDLINE until April 13, 2023, and included 24 studies (98 articles) evaluating the impact of community-based infant/child food interventions/supplements for the prevention of wasting among children up to 5 years.

**Data extraction:**

Two review authors independently extracted data and assessed the quality of included studies using the Cochrane Risk of Bias Tool 2.0. Grading of Recommendations, Assessment, Development, and Evaluation (GRADE) criteria were used to assess the quality of evidence.

**Data analysis:**

This review included 19 cluster–randomized controlled trials (cRCTs) and 5 RCTs evaluating the impact of community-based infant/child food interventions/supplements including fortified blended foods (FBFs), small-quantity (SQ), medium-quantity (MQ), or large-quantity (LQ) lipid-based nutrient supplements (LNS), and multiple micronutrient powder (MNP) for the prevention of wasting among children up to 5 years of age. The analysis showed that infants/children given supplementation with LNS (either SQ, MQ, or LQ) had significantly reduced wasting and significant improvements in weight-for-age z-score, mid–upper-arm circumference (MUAC), and underweight prevalence, along with significant reductions in mortality. The MNP supplementation had little or no impact on wasting but was associated with increased incidences of rapid breathing/chest indrawing and diarrhea morbidity. Overall, the studies were judged to have raised some concerns for the outcomes of wasting and adverse anthropometric indices. However, the GRADE analysis suggested low-to-moderate certainty of outcomes.

**Conclusions:**

The findings of this review highlight the effectiveness of SQ-LNS and MQ/LQ-LNS supplementation in decreasing rates of wasting, underweight, and mortality and increasing MUAC and weight-for-age z-scores. Methodological limitations in most studies emphasize the need for future trials with direct comparisons of various dietary supplementation strategies.

**Systematic review registration:**

PROSPERO registration number CRD42021277429.

## INTRODUCTION

Malnutrition in children remains a pressing concern, impeding the growth and development of children and predisposing them to future health complications. In 2020, approximately 38 million children were overweight, 149 million had stunted growth, and 45 million suffered wasting.^1^ Wasting is defined as a low–weight-for-height *z*-score (WHZ) and is further classified as moderate or severe.^2–4^ In 2018, 16.6 million children suffered from severe acute malnutrition,^1^ with wasting contributing to 12.6% of the global mortality in children younger than 5 years.^5^ Despite several interventions, global wasting prevalence has declined slowly, with just 19% of countries on track to meet the World Health Assembly 2025 targets of maintaining the prevalence of wasting below 5.0%.^5^ Regional disparities persist, with over half (25 million) of the children with wasting residing in South Asia.^1^ Additionally, three-quarters of all children suffering from severe wasting reside in Asia.

Childhood wasting is influenced by several factors, including maternal malnutrition, which may contribute to low birth weight and prematurity. Other factors may include inadequate infant and young child feeding (IYCF) practices, limited healthcare access, famine and food insecurity, and unsanitary environments.^6^ Wasting is also associated with infectious diseases,^7^ amplifying mortality risks from conditions like diarrhea and pneumonia. While often considered an acute issue, wasting may have consequences that extend beyond early childhood, encompassing long-term cognitive and developmental challenges, immune system impairment, and increased susceptibility to noncommunicable diseases later in life.^1^ It is estimated that mortality rates among children with severe wasting are 9-fold higher than those of normal-weight counterparts.^8^

Preventing wasting necessitates comprehensive strategies, beginning with maternal health and nutrition, antenatal care, household food security, food and market accessibility, healthcare accessibility, and water, sanitation, and hygiene practices.^9^ The critical first 1000 days from conception through infancy and early childhood offer a window of opportunity to establish a foundation for lifelong health and development.^10^ Interventions like community-based maternal and child nutritional supplementation, breastfeeding promotion, and infant nutrition education for infants in low-to-middle–income countries (LMICs) show promise in reducing the risk of wasting.^11^ Despite global nutrition efforts primarily focusing on the prevention of stunting and the treatment of wasting, the scarcity of robust studies on interventions to prevent wasting persists.^12^ Addressing this gap is vital to advancing global nutrition agendas and enhancing the effectiveness of community-based preventive measures in combating wasting.

### Objective

In this systematic review we aimed to evaluate the effectiveness of community-based infant/child food interventions and supplements (including fortified blended foods [FBFs]; lipid-based nutrient supplements [LNS] in small quantities [SQ-LNS], medium quantities [MQ-LNS], and large-quantities [LQ-LNS]; and multiple micronutrient powders [MNPs]) in preventing wasting among children up to 5 years at risk of wasting and nutritional oedema. This review was commissioned by the World Health Organization (WHO) to update their guidelines on childhood wasting.

## METHODS

The protocol for this review has been registered with the International Prospective Register of Systematic Reviews (PROSPERO registration No. CRD42021277429). Preferred Reporting Items for Systematic Reviews and Meta-Analyses (PRISMA) guidelines were followed for reporting the results of the systematic review (File S1)

### Scope of the WHO Guidelines

The systematic reviews for the WHO guidelines focused on studies of prevention interventions that reported the prevalence/incidence of wasting or deterioration to severe wasting. These were the critical outcomes prioritized by the guideline development group.

These prevention interventions included community-based interventions and strategies for preventing wasting in infants and children, which were defined as single and/or multicomponent approaches aimed primarily at preventing wasting in children up to 5 years and delivered within an entire community or a large part of a community. Studies on prevention and treatment were included if the participants and their outcomes were separately reported. Based on these criteria, the systematic reviews identified the following 5 broad categories:

- Food-based interventions/supplements
- Education and counseling
- Financial relief interventions
- Agricultural interventions
- Other interventions

Out of these 5 broad categories, only the interventions expected to have a direct impact on improving access to nutrient-dense foods or improving overall child nutrition were considered comparisons of interest for this guideline. These interventions fell under 3 broad categories: (1) food-based interventions/supplements; (2) intensive IYCF counseling; and (3) financial relief interventions. In this article, we report findings on community-based food interventions/supplements for infants/children at risk of wasting and nutritional oedema. The findings on maternal food intervention/supplementation, IYCF counseling, and financial relief interventions will be published separately.

### Eligibility Criteria

Studies reporting on community-based child food interventions/supplements (including MMPs) targeting children up to 5 years old at risk of wasting and nutritional oedema were included in Table 1. All eligible interventions included community-based food interventions/supplements, defined as single- or multi-component approaches primarily aimed at preventing wasting in children up to 5 years old, delivered within an entire community or a large part of it. Community-based food interventions/supplements included interventions that may have impacts on outcomes of growth and wasting in infants and children, such as the interventions included.

**Table 1.** PICOS criteria for inclusion and exclusion of studies.

*Inclusion criteria*
Participants: Infants and children (up to 5 y old) at risk of wasting and nutritional oedema. Evidence from a broader age group but inclusive of our target population was included the outcome was reported separately for children up to 5 y old.
Intervention: Community based nutritional supplementation, including FBFs; SQ-, MQ-, and LQ-LNS; and MNP.
Comparison: No intervention or standard of care.
Primary outcomes: Childhood wasting and deterioration to severe wasting
Secondary outcomes: Anthropometric outcomes (including WHZ, MUAC, WAZ, and underweight), morbidity, and mortality. We only included studies reporting at least 1 of the primary outcomes of interest.
Study design: RCTs (individually or cluster)
*Exclusion criteria*
Studies including critically ill children or children with a pre-existing health condition (such as cancer, diabetes, metabolic disorders, congenital abnormalities, etc.)

*Abbreviations:* FBFs, fortified blended foods; LNS, lipid-based supplementation; LQ, large quantity; MNP, multiple micronutrient powder; MQ, medium quantity; MUAC, mid–upper arm circumference; PICOS, Population, Intervention, Comparison, Outcome, and Study design; RCT, randomized controlled trial; SQ, small quantity; WAZ, weight for age *z*-score; WHZ, weight for height *z*-score.

- Food-based interventions/supplements for infants/children:
  - Fortified blended foods (FBFs): Blends of partially precooked and milled cereals, soya, beans, and pulses fortified with micronutrients (vitamins and minerals), typically provided as Corn Soy Blend (CSB) or Wheat Soy Blend (WSB).
  - Lipid-based-nutrient supplementation (LNS):
    - Medium-quantity LNS (MQ-LNS) and large-quantity LNS (LQ-LNS): Defined based on the caloric content of the product, as specified in the World Food Program (WFP) specifications (ie, MQ-LNSs are LNSs intended to provide approximately 250-499 kcal/d and LQ-LNSs are LNSs intended to provide >500 kcal/d).
    - Small-quantity LNS (SQ-LNS): Provide approximately 100-120 kcal/d.
  - Child MNPs: Defined as MNPs containing >3 micronutrients, excluding multivitamins.
- Food-based interventions/supplements for infants/children in addition to mothers:
  - FBFs
  - SQ-LNS

Studies that provided both preventive and therapeutic supplementation were eligible for inclusion if the outcomes were separately reported for preventive supplementation, excluding studies focusing solely on wasting treatment. Studies comparing community prevention interventions with no intervention or standard of care, or studies in which the intervention and comparison arms had the same co-interventions, were included. However, studies comparing 2 different types of preventive interventions/strategies were excluded.

The study population comprised children up to 5 years old at risk of wasting and nutritional oedema, excluding those with pre-existing health conditions (such as cancer, diabetes, metabolic disorders, congenital abnormalities, etc), except for infants/children with HIV. Additionally, studies focusing solely on critically ill and hospitalized infants/children were excluded. The definition of infants at risk of wasting and nutritional oedema is given in File S2.

Studies reporting data on at least 1 of the primary outcomes, which comprised wasting incidence and prevalence and deterioration to severe wasting, were included. Secondary outcomes included other anthropometric outcomes (such as WHZ, mid–upper-arm circumference [MUAC], weight-for-age *z*-score [WAZ], and underweight), morbidity (such as diarrhea, fever, cough or respiratory illnesses, etc), and mortality. The criteria for considering studies for this review are detailed in Table 1.

### Search Methods and Study Selection

The search strategy was developed using Medical Subject Heading (MeSH) terms and keywords. Searches were run on the following electronic databases: in The Cochrane Library the Cochrane Database of Systematic Reviews (CDSR) and the Cochrane Central Register of Controlled Trials (CENTRAL); The Campbell Library; MEDLINE; EMBASE; CINAHL; SCOPUS; Web of Science; and eLANA (WHO). Additionally, the bibliography of included studies and relevant systematic reviews was screened to identify any studies missed during the initial electronic search. The initial search on the above 9 databases was searched until July 2021 and an updated search on MEDLINE and Ovid MEDLINE was conducted until April 13, 2023 (File S3). To retain a broader search, no limits were applied to date, language, or outcomes.

Studies identified through the electronic search were exported into EndNote and then uploaded and de-duplicated into Covidence, a web-based systematic review software designed for screening.^13^ Two reviewers (K.A.R. and A.A.) independently assessed all titles, abstracts, and full texts for potential inclusion based on the eligibility criteria. Discrepancies were resolved through discussion until a consensus was reached or by involving a third author (Z.S.L. or J.K.D.). Reasons for exclusion were documented for all studies excluded during the full-text screening phase.

### Data Extraction

Two reviewers (K.A.R. and Z.A.P.) independently extracted data on a pretested extraction sheet in Excel. The data extracted included study and participant characteristics, intervention and control details, delivery platform, contextual factors, and outcomes. Discrepancies in the data extraction process were resolved through discussion until consensus was reached, or a third reviewer (Z.S.L.) was consulted if required. Efforts were made to contact the authors of the included studies for clarifications or additional data.

### Statistical Analysis

The meta-analysis was performed using Review Manager (RevMan) Web. For dichotomous outcomes, results were presented as risk ratios (RRs) with 95% CIs, while continuous outcomes were presented as mean differences (MDs) with 95% CIs. A random-effects model was employed to accommodate expected heterogeneity in study settings, exposures, comparisons, and outcomes across studies. Median and interquartile range data were converted to means and SDs using standard formulas. Adjusted data were utilized when pooling data from individual and cluster-randomized controlled trials (c-RCTs). In instances where adjusted results were unavailable for c-RCTs, the data were adjusted by calculating the design effect using intercluster coefficients (ICCs) reported in the study or by extracting ICCs from a similar study. Statistical heterogeneity was evaluated using *I*^2^ and the significance of the χ^2^ test, along with visual inspection of forest plots.

### Risk of Bias Assessment and Evidence Profiles

The quality assessments of included studies were carried out by use of the updated Cochrane risks of bias (ROB) tool ROB-2, which was designed for RCTs.^14^ Two independent authors (K.A.R., Z.A.P.) assessed the quality of all eligible studies, with discrepancies resolved through consensus or by contacting a third author (Z.S.L.) when necessary. The ROBs in the trial were judged as “high,” “low,” or “some concerns” (ie, when the trial was judged to raise some concerns in at least 1 domain, but not to be at high ROB for any domain).

To evaluate the certainty of evidence, Grading of Recommendations, Assessment, Development, and Evaluation (GRADE) criteria were used to construct GRADE evidence profiles for all outcomes.^15^ The GRADE criteria are used to assess the evidence based on the risk of bias, inconsistency, indirectness, imprecision, and risk of publication bias. Certainty of the evidence for each outcome was rated as “high” (indicating high confidence in results), “moderate” (indicating moderate confidence in effect estimate), “low” (indicating limited confidence in effect estimate), or “very low” (indicating very little or no confidence in the effect estimates).

## RESULTS

### Search Results

**Figure 1.**
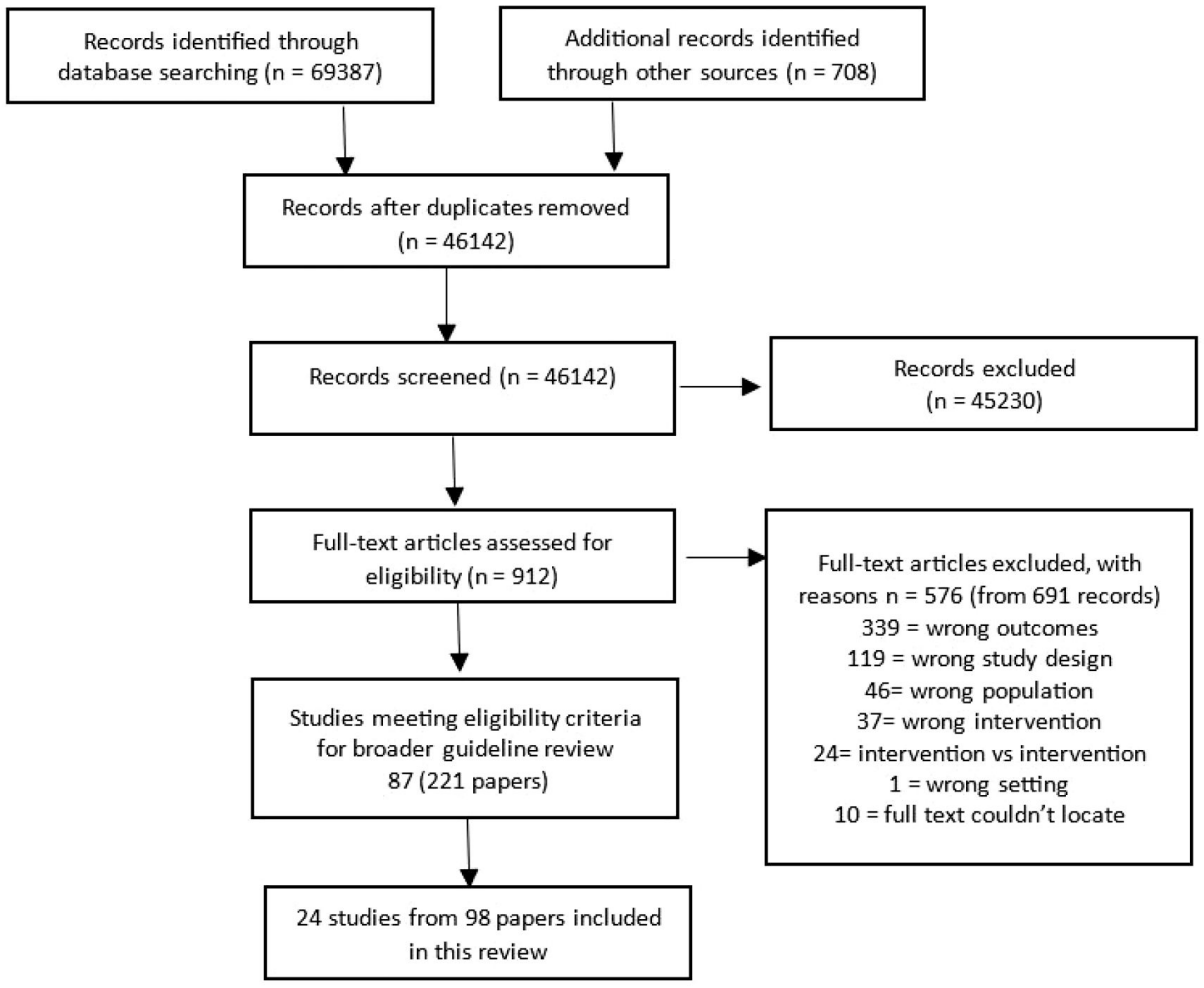
PRISMA Diagram.

A total of 69 387 articles were initially identified through the database search, with an additional 708 articles identified through cross-referencing and a grey literature search. After the removal of duplicates, 46 142 articles underwent title and abstract screening, which was followed by a full-text screening of the remaining 912 studies. A total of 24 studies (98 articles) focusing on infant/child intervention/supplementation were included in the final review (Figure 1).^16–113^ All of the included trials were meta-analyzed. Of the included trials, 20 trials (from 21 articles) focused on infant/child supplementation,^32^^,^^37^^,^^44^^,^^46^^,^^49^^,^^51^^,^^53–55^^,^^63^^,^^73–75^^,^^79^^,^^83^^,^^84^^,^^89^^,^^92^^,^^93^^,^^99^^,^^100^ 4 reported on both infant and child supplementation in addition to supplementation for mothers during pregnancy,^23^^,^^28^^,^^46^^,^^71^ and 1 multiple-arm study overlapped both categories.^46^

### Characteristics and Quality of Included Studies

Among the trials on infants and children that focused on food intervention/supplementation only to infants and children, data were provided on FBF in 4 trials,^37^^,^^44^^,^^75^^,^^84^ on MQ-LNS/LQ-LNS in 8 trials,^37^^,^^44^^,^^53^^,^^55^^,^^63^^,^^74^^,^^75^^,^^93^ on SQ-LNS in 9 trials,^32^^,^^46^^,^^49^^,^^51^^,^^54^^,^^73^^,^^74^^,^^79^^,^^89^ and on multiple micronutrient supplementation to infants/children in 6 trials.^37^^,^^46^^,^^83^^,^^92^^,^^99^^,^^100^ Among the trials on infants/children that also focused on maternal food intervention/supplementation, 3 trials provided data on SQ-LNS^23^^,^^28^^,^^46^ and 1 trial provided data on FBF supplementation to both infants and children and their mothers during pregnancy.^71^

Of the included trials, 19 were c-RCTs^32^^,^^37^^,^^44^^,^^46^^,^^49^^,^^51^^,^^53–55^^,^^63^^,^^71^^,^^73^^,^^79^^,^^83^^,^^84^^,^^89^^,^^92^^,^^93^^,^^99^ and 5 were individual RCTs.^23^^,^^28^^,^^74^^,^^75^^,^^100^ Most of the trials were from the African region (*n* = 13),^23^^,^^28^^,^^32^^,^^48^^,^^51^^,^^53–55^^,^^71^^,^^74^^,^^75^^,^^79^^,^^89^ 5 were from the Southeast Asia region,^44^^,^^46^^,^^73^^,^^83^^,^^99^ 3 were from the Eastern Mediterranean region,^63^^,^^92^^,^^93^ and 3 were from the Western Pacific region.^37^^,^^84^^,^^100^ More information on child age and duration of supplementation is provided in Table 2.^16–113^

**Table 2.** Characteristics of included studies.

Author	Country	Study design	FBF arms	Comparison arms
**Community-based interventions**				
**FBF infant/child**				
Borg et al. (2020)^36^^,^^37^	Cambodia	cRCT	CSB++ given for 1 mo Amount: 250-500 kcal/d	No intervention
Christian et al. (2015)^26^^,^^38–40^^,^^43^^,^^44^^,^^90^	Bangladesh	cRCT	WSB++ given for 12 mo Amount: 125 kcal/d for infants 6-12 mo old; 250 kcal/d for children 12-18 mo old All study arms received nutritional counseling	Nutritional counseling alone
Mangani et al. (2015)^16^^,^^75^	Malawi	RCT	Micronutrient-fortified CSB given for 12 mo Amount: 284 kcal/d (71 g/d)	No intervention
Pham et al. (2012)^84^^,^^98^	Vietnam	cRCT	Locally produced micronutrient-fortified complementary foods (instant flour [FF] or an food complement [FC]) given for 6 mo Amount: 100 kcal/100 g; 270 g/d consumed	Traditional complementary foods (gruel)
**LQ-LNS and MQ-LNS – infant/child**				
Borg et al. (2020)^36^^,^^37^	Cambodia	cRCT	Locally produced fish-based RUSF (MQ-LNS) given for 6 mo to infants 6-11 mo old at baseline Amount: 250 to 500 kcal/d (40-110 g/d)	No intervention
Christian et al. (2015)^26^^,^^38–40^^,^^43^^,^^44^^,^^90^	Bangladesh	cRCT	Plumpy’doz, chickpea-based ready-to-use food, and rice-lentil ready-to-use food (all MQ-LNS) given for 12 mo to infants 6 mo old at baseline Amount: 125 kcal/d for infants 6-12 mo old and 250 kcal/d for children 12-18 mo old All study arms received nutritional counseling	Nutritional counseling alone
Mangani et al. (2015)^16^^,^^75^	Malawi	RCT	Milk-LNS and soy-LNS (MQ-LNS) given for 12 mo to infants 5.5-6.5 mo old at baseline Amount: 285 kcal/d (54 g/d) (milk-LNS) and 276 kcal/d (54 g/d) (soy-LNS)	No intervention
Huybregts et al. (2012)^53^	Chad	cRCT	Plumpy’Doz (MQ-LNS) given for 4 mo to infants and children 6-36 mo old at baseline Amount: 247 kcal/d (46 g/d) All arms received a monthly food package	Monthly food package
Isanaka et al. (2009)^55^^,^^56^	Niger	cRCT	RUTF (LQ-LNS) given for 3 mo to infants and children 6-60 mo old at baseline Amount: 500 kcal/d (92 g/d)	No intervention
Khan et al. (2020)^63^	Pakistan	cRCT	Locally-produced MQ-LNS given for at least 6 mo to infants and children 6-18 mo old at baseline Amount: 255-280 kcal/d (50 g/d) Health and hygiene messages were provided	No intervention Routine public and private health services available within the area
Maleta et al. (2015)^33^^,^^66^^,^^74^	Malawi	RCT	No-milk LNS (40 g/d) and milk-LNS (40 g/d) (MQ-LNS) given for 12 mo to infants 6 mo old at baseline Amount: 241 kcal/d (40 g/d)	No intervention
Soofi et al. (2022)^62^^,^^93^	Pakistan	cRCT	1. Locally produced LNS in medium quantity (called Wawamum) was provided to children from 6 to 24 mo old for 18 mo (combined with UCT) Amount: 50 grams of Wawamum (1 sachet) received sachets on monthly basis 2. LNS (combined with UCT and SBCC)	UCT only UCT + SBCC
**SQ-LNS infant/child**				
Maleta et al. (2015)^33^^,^^66^^,^^74^	Malawi	RCT	No-milk LNS (20 g/d) and milk-LNS (20g/d) were given for 12 mo to infants 6 mo old at baseline Amount: 117 kcal/d (20 g/d)	No intervention
Becquey et al. (2019)^32^	Burkina Faso	cRCT	SQ-LNS (20 g/d) given monthly from age 6 to 23.9 mo Amount: 118 kcal/d (20 g/d) Enhanced BCC also provided in addition to SQ-LNS related BCC	Nutrition, hygiene, and health BCC
Huybregts et al. (2019)^52^^,^^54^	Mali	cRCT	SQ-LNS (20 g/d) given monthly from 6 to 23.9 mo old Amount: 118 kcal/d (20 g/d) Enhanced BCC provided in addition to SQ-LNS related BCC	Nutrition, hygiene, and health BCC
Dewey et al. (2017)^20^^,^^45^^,^^46^^,^^76^^,^^77^^,^^96^^,^^97^	Bangladesh	cRCT	SQ-LNS (20 g/d) given to infants 6 mo old at baseline for 18 mo Amount: 118 kcal/d (20 g/d) IFA given to caregivers	No intervention for children IFA given to caregivers
Hess et al. (2015)^17–19^^,^^48–50^^,^^85^^,^^87^^,^^91^	Burkina Faso	cRCT	SQ-LNS (containing 5mg Zn or 10mg Zn) given for 9 mo to infants 6 mo old at baseline Amount: 118 kcal/d (20 g/d)	No intervention
Humphrey et al. (2019) & Prendergast et al. (2019) (Shine Trial)^51^^,^^89^^,^^95^	Zimbabwe	cRCT	SQ-LNS (plus WASH) for 12 mo to infants 6 mo old at baseline With IYCF counseling Amount: 118 kcal/d (20 g/d) SQ-LNS only for 12 mo to infants 6 mo old at baseline With IYCF counseling Amount: 118 kcal/d (20 g/d) *disaggregated by children born to HIV-negative women (Humphrey 2019) and HIV-exposed children (Prendergast 2019)	1) WASH only 2) Standard of care counseling *disaggregated by children born to HIV-negative women (Humphrey 2019) and HIV-exposed children (Prendergast 2019)
Luby et al. (2018)^27^^,^^73^	Bangladesh	cRCT	SQ-LNS (plus WASH) for 18 mo to infants 6 mo old at baseline With IYCF counseling Amount: 118 kcal/d (20 g/d) SQ-LNS only for 18 mo to infants 6 mo old at baseline With IYCF counseling Amount: 118 kcal/d (20 g/d)	1) WASH only 2) No intervention
Null et al. (2018)^79^	Kenya	cRCT	SQ-LNS (plus WASH) for 18 mo to infants 6 mo old at baseline With IYCF counseling Amount: 118 kcal/d (20 g/d) SQ-LNS only for 18 mo to infants 6 mo old at baseline With IYCF counseling Amount: 118 kcal/d (20 g/d)	1) WASH only 2) No intervention
**SQ-LNS maternal and infant/child**				
Dewey et al. (2017)^20^^,^^45^^,^^46^^,^^76^^,^^77^^,^^96^^,^^97^	Bangladesh	cRCT	SQ-LNS given to mothers in pregnancy and for 6 mo postpartum. Amount:118 kcal/d (20 g/d) SQ-LNS given to infants from 6 mo old for 18 mo Amount: 118 kcal/d (20 g/d)	No intervention for children IFA given to mothers in pregnancy and for 3 mo postpartum
Adu-Afarwuah et al. (2016)^20^^,^^23–25^	Ghana	RCT	SQ-LNS given to women during pregnancy and for 6 mo postpartum Amount:118 kcal/d (20 g/d) SQ-LNS given to infants from 6 mo old for 12 mo Amount:118 kcal/d (20 g/d)	No intervention for children IFA given to mothers in pregnancy and placebo for 6 mo postpartum MMN given to mothers in pregnancy and for 6 mo postpartum
Ashorn et al. (2015)^21–23^^,^^28–31^^,^^34^^,^^35^^,^^41^^,^^42^^,^^47^^,^^57^^,^^58^^,^^60^^,^^64^^,^^65^^,^^78^^,^^80^^,^^81^^,^^86^^,^^88^^,^^94^	Malawi	RCT	SQ-LNS given to women during pregnancy and for 6 mo postpartum Amount:118 kcal/d (20 g/d) SQ-LNS given to infants from 6 mo old for 12 mo Amount:118 kcal/d (20 g/d)	No intervention for children IFA given to mothers in pregnancy and placebo for 6 mo postpartum MMN given to mothers in pregnancy and for 6 mo postpartum
**FBFs – maternal & infant/child**				
Leroy et al. (2021)^69–72^^,^^82^	Burundi	cRCT	Corn-soy blend and micronutrient-fortified vegetable oil given to mothers during pregnancy up to 6 mo postpartum and children 6-24 mo old (T24 arm) or from pregnancy up to 6 mo postpartum and children 6-18 mo old (T18 arm) Amount: The child ration provided 458 kcal/d (3 kg of CSB and 300 g of oil) per month The maternal ration during pregnancy and 6 mo postpartum included 6 kg of CSB and 600 g of oil per month The household food ration included 12 kg of CSB and 1200 g of oil per month BCC also given	No rations or BCC Access to the standard health-care services provided by the Ministry of Health
**MNPs – infant/child**				
Borg et al. (2020)^36^^,^^37^	Cambodia	cRCT	MNPs were given for 6 mo to infants 6-11 mo old at baseline	No supplementation
Barffour et al. (2019)^100–113^	Central LaoPeople’s Democratic Republic (PDR)	RCT	High-zinc, low-iron micronutrient powder (10 mg/d zinc, 6 mg/d iron, and 13 other micronutrients) was given to children aged 6-23 mo	Daily placebo powder
Dewey et al. (2017)^20^^,^^45^^,^^46^^,^^76^^,^^77^^,^^96^^,^^97^	Bangladesh	cRCT	MNPs were given to infants 6 mo old until they reached 24 mo old Mothers in the intervention and control arms received IFA daily during pregnancy and every alternate day during the first 3 mo postpartum	No supplementation for children Mothers in the intervention and control arms received IFA daily during pregnancy and every alternate day during the first 3 mo postpartum
Osei et al. (2015)^83^	Nepal	cRCT	MNPs were given to infants 6-9 mo old at baseline for 11 mo EHFP was also delivered to intervention and comparison arms	Enhanced homestead food production (EHFP) Home gardens, poultry and nutrition education
Soofi et al. (2013)^92^	Pakistan	cRCT	MNPs were given to infants 6 mo old at baseline until 18 mo old The MNP with Zn arm was used for this analysis	No supplementation
Young et al. (2021)^61^^,^^67^^,^^68^^,^^99^	India	cRCT	MNPs were given for 12 mo to infants and children 6-18 mo old at baseline IYCF counseling also delivered	No supplementation IYCF counseling only

Abbreviations: EHFP, enhanced homestead food production; IYCF, infant and young child feeding; SBCC, social and behavior change communication; UCT, unconditional cash transfer; WASH, waste, sanitation, and hygiene.

* Maternal interventions within scope include those given during pregnancy.

**Figure 2.**
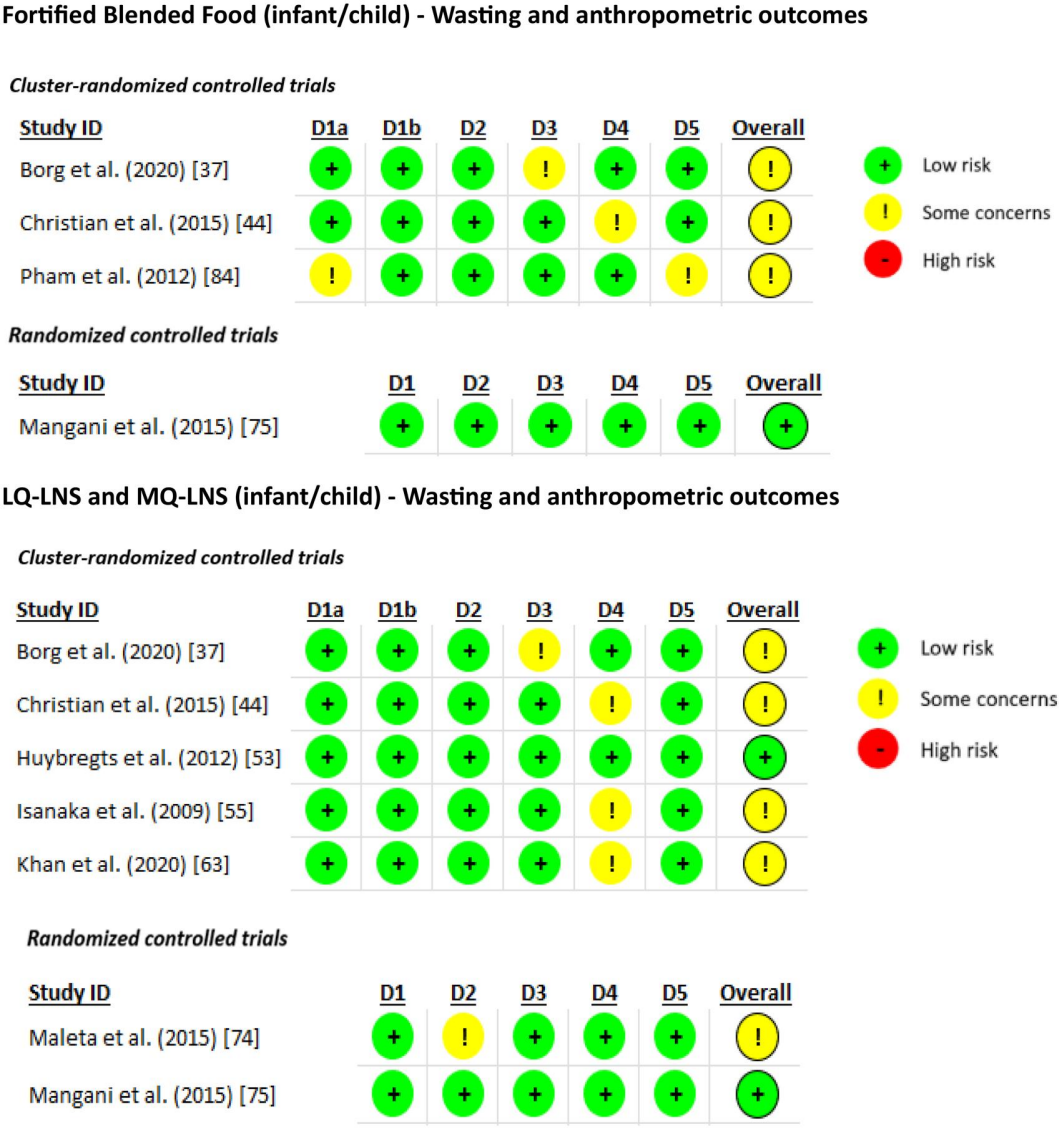

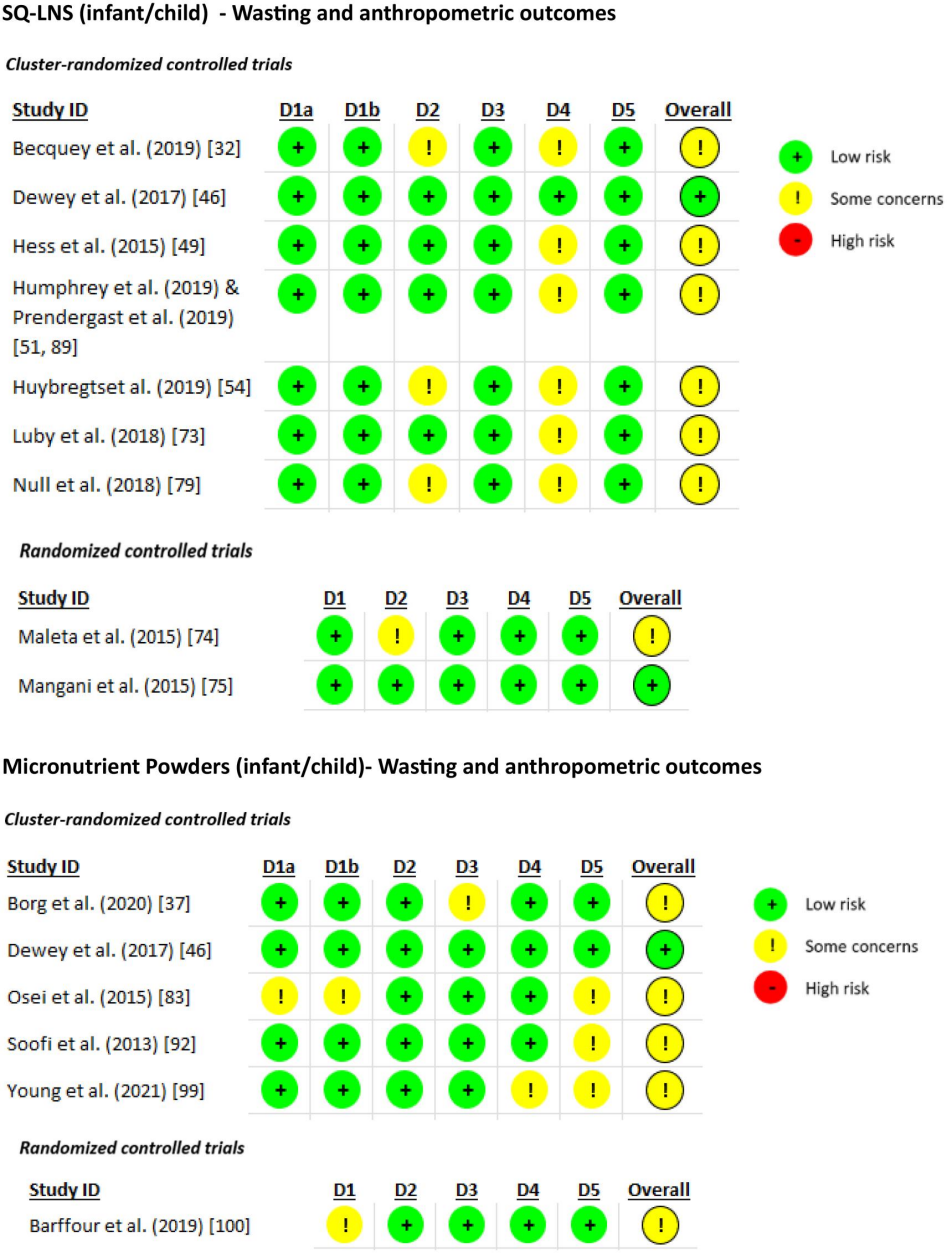

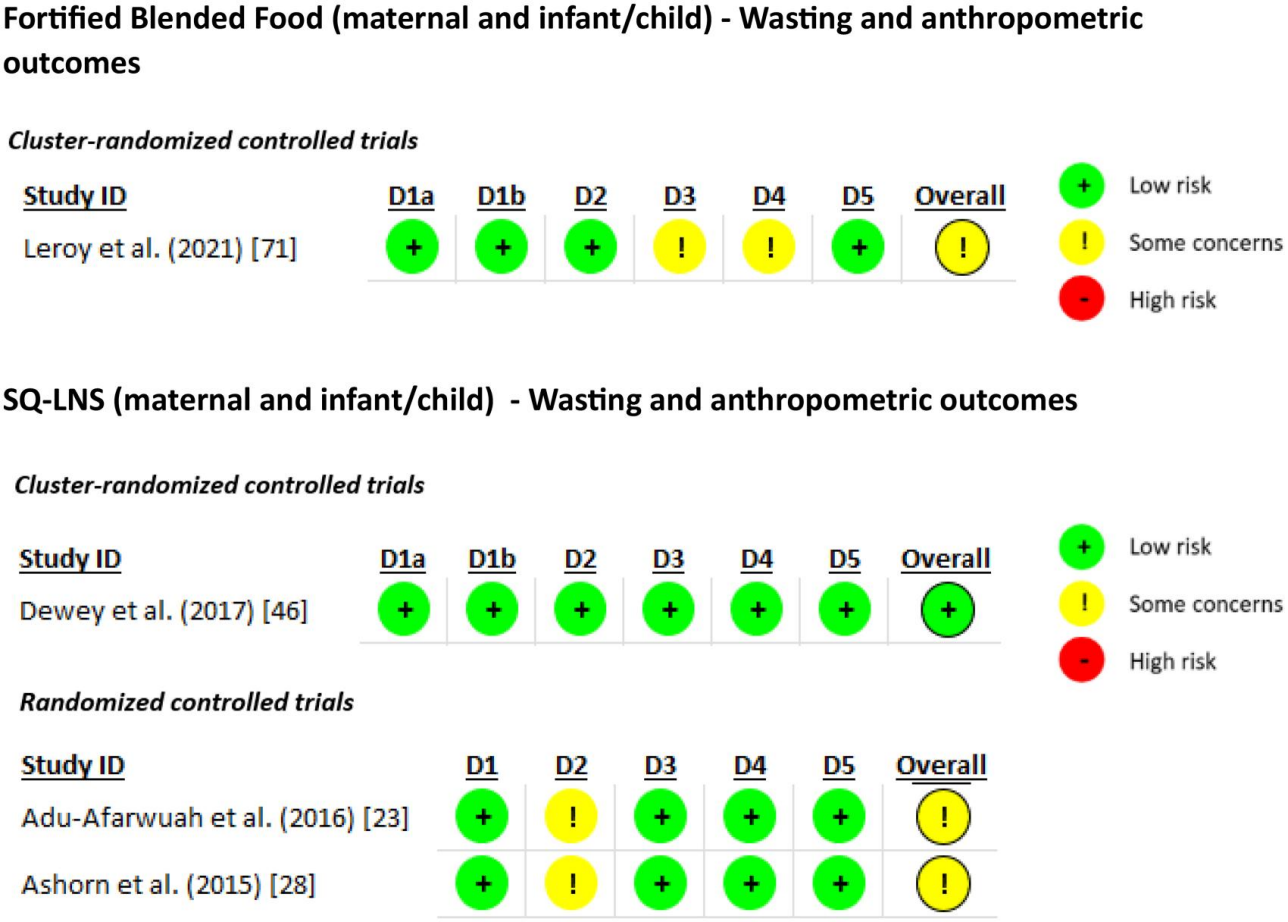
Risk of Bias Assessment by Wasting and Anthropometric Outcomes.

Overall, the studies were judged to have some concerns regarding the outcomes of wasting and anthropometry. The detailed risk of bias rankings of individual trials based on wasting and anthropometric outcomes is given in Figure 2,^23^^**,**^^28^^**,**^^32^^**,**^^37^^**,**^^44^^**,**^^46^^**,**^^49^^**,**^^51^^**,**^^53–55^^**,**^^63^^**,**^^71^^**,**^^73–75^^**,**^^79^^**,**^^83^^**,**^^89^^**,**^^92^^**,**^^99^^**,**^^100^ and the risk of bias ranking of individual trials based on morbidity and mortality outcomes is given in File S4. The effect of community-based food interventions/supplements for infants/children is summarized in Table 3.

**Table 3.** Effect estimates of the infant/child supplementation.

Interventions	Infant/child supplementation				Maternal/child supplementation	
Outcomes	FBF	LQ-LNS and MQ-LNS	SQ-LNS	MNP	SQ-LNS	FBF
Prevalence of wasting	RR 1.05; 95% CI, 0.83 to 1.33; 3 trials; I^2^ 0%	RR 0.87; 95% CI, 0.74 to 1.03; four trials: I^2^ 0%	RR 0.88; 95% CI, 0.79 to 0.98; eight trials; I^2^ 0%	RR 1.01: 95% CI, 0.84 to 1.21; 6 trials; I^2^ 41%	RR 0.86; 95% CI, 0.70 to 1.06; 3 trials; I^2^ 0%	T18 arm: RR 0.51; 95% CI, 0.31 to 0.84; 1 trial T24 arm: RR 0.70; 95% CI, 0.40 to 1.21; 1 trial
Prevalence of moderate wasting			RR 0.98; 95% CI, 0.82 to 1.18; two trials; I^2^ 0%			
Incidence of wasting		RR 0.74; 95% CI, 0.55 to 0.99; two trials; I^2^ 44.5%	RR 0.76; 95% CI, 0.57 to 1.02; two trials; I^2^ 0%			
Cumulative incidence of wasting		RR 0.77: 95% CI, 0.53 to 1.12; 3 trials; I^2^ 0%	RR 0.94; 95% CI, 0.58 to 1.52; 1 trial			
Prevalence of severe wasting	RR 1.53; 95% CI, 0.26 to 9.07; 1 trial	RR 0.73; 95% CI 0.12 to 4.32 to 1 trial	RR 0.78; 95% CI, 0.45 to 1.33; two trials; I^2^ 9%		RR 2.07; 95% CI, 0.42 to 10.15; 1 trial	
Cumulative incidence of severe wasting	RR 1.50; 95% CI, 0.25 to 8.88; 1 trial	RR 0.55; 95% CI, 0.32 to 0.92; 3 trials; I^2^ 23.2%	RR 1.82; 95% CI, 0.51 to 6.47; 1 trial			
WHZ	MD: 0.02; 95% CI, −0.02 to 0.05; four trials; I^2^ 0%	MD 0.03; 95% CI, −0.04 to 0.11; seven trials; I^2^ 64.3%	MD 0.08; 95% CI, 0.03 to 0.12; nine trials; I^2^ 44%	MD 0.02; 95% CI, −0.04 to 0.07; five trials; I^2^ 0%	MD 0.05; 95% CI, −0.03 to 0.14; 3 trials; I^2^ 34%	T18 arm: MD 0.2; 95% CI, 0.05 to 0.35; 1 trial T24 arm: MD 0.1; 95% CI, −0.07 to 0.27; 1 trial
MUAC (cm)	0.07 cm; 95% CI, −0.05 to 0.19; two trials; I^2^ 15%	MD 0.14 cm; 95% CI, 0.08 to 0.2; four trials; I^2^ 0%	MD 0.13 cm; 95% CI, 0.00 to 0.26; four trials; I^2^ 80%	MD 0 cm; 95% CI, −0.09 to 0.09; two trials; I^2^ 0%	MD 0.01 cm; 95% CI, −0.18 to 0.12; two trials; I^2^ 64%	
MUACZ			MD 0.06; 95% CI, 0 to 0.11; four trials; I^2^ 0%	MD −0.05; 9% CI, −0.13 to 0.03; 1 trial	MD 0.02; 95% CI, −0.05 to 0.09; two trials; I^2^ 0%	
WAZ	MD 0.07; 95% CI, −0.04 to 0.18; four trials; I^2^ 54%	MD 0.03; 95% CI, 0.00 to 0.06; five trials; I^2^ 0%	MD 0.14; 95% CI, 0.09 to 0.19; seven trials; I^2^ 29%	MD 0.01; 95% CI −0.08 to 0.09; five trials; I^2^ 63%	MD 0.10; 95% CI, −0.03 to 0.23; 3 trials; I^2^ 70%	
Prevalence of underweight	RR 0.97; 95% CI, 0.71 to 1.32; 3 trials; I^2^ 25%	RR 0.91; 95% CI, 0.83 to 1.00; 4 trials; I^2^ 0%	RR 0.84; 95% CI, 0.78 to 0.90; 7 trials; I^2^ 0%	RR 1.01; 95% CI, 0.94 to 1.08; 6 trials; I^2^ 0%	RR 0.93; 95% CI, 0.83 to 1.05; 3 trials; I^2^ 0%	
Mortality	RR 0.97; 95% CI, 0.47 to 2.00; 2 trials; I^2^ 0%	RR 0.60; 95% CI, 0.37 to 0.98; 7 trials; I^2^ 76.8%	RR 0.76; 95% CI, 0.63 to 0.91; 9 trials; I^2^ 0%	RR 1.04; 95% CI 0.35 to 3.05; 3 trials; I^2^ 55%	RR 0.62; 95% CI, 0.24 to 1.58; 3 trials; I^2^ 39%	
Incidence of diarrhea	RR 0.97: 95% CI, 0.83 to 1.13; 1 trial	RR 0.97: 95% CI, 0.90 to 1.04; 3 trials; I^2^ 0%	RR 0.94; 95% CI, 0.80 to 1.11; five trials; I^2^ 49%	RR 1.12; 95% CI, 1.00 to 1.25; 1 trial	RR 0.59; 95% CI, 0.20 to 1.79; 1 trial (requiring hospitalisation)	
Prevalence of diarrhea				RR 0.72; 95% CI, 0.60 to 0.88; 3 trials; I^2^ 0%	RR 1.60; 95% CI 0.84 to 3.04; 1 trial	
Incidence of pneumonia	RR 0.89; 95% CI 0.69 to 1.14; 1 trial				RR 0.71; 95% CI, 0.42 to 1.19; 1 trial (requiring hospitalisation)	
Incidence of pneumonia or respiratory infections		RR 0.92; 95% CI, 0.80 to 1.07; 3 trials; I^2^ 66.9%				
Prevalence of cough, or respiratory infections			RR 0.96; 95% CI, 0.28 to 3.31; two trials; I^2^ 0%			
Prevalence of ARI			RR 1.12; 95 % CI, 0.77 to 1.64; 1 trial	Acute lower RI: RR 0.85; 95% CI, 0.57 to 1.26; 1 trial	RR 0.96; 95% CI, 0.65 to 1.39; 1 trial	
Incidence of rapid breathing or chest indrawing				RR 1.61; 95% CI, 1.32 to 1.96; 1 trial		
Prevalence of high fever			RR 1.04; 95% CI, 0.80 to 1.34; 1 trial		RR 0.83; 95% CI, 0.64 to 1.08; 1 trial	
Incidence of fever				RR 0.95; 95% CI, 0.73 to 1.23; 1 trial		

### Effect of Intervention

#### Infant/Child Food Interventions/Supplements

*Infant/child supplementation with FBFs:* Four trials^37^^,^^44^^,^^75^^,^^84^ reported on FBF supplementation to infants/children. Trials provided FBF in the form of Fortified Corn-Soy Blend (CSB++),^37^ Fortified Wheat Soy Blend (WSB++),^44^ and micronutrient-fortified CSB^75^ as locally produced micronutrient-fortified complementary foods.^84^ The duration of the interventions ranged between 6 and 12 months. The full evidence profiles can be found in File S5.

**Figure 3.**
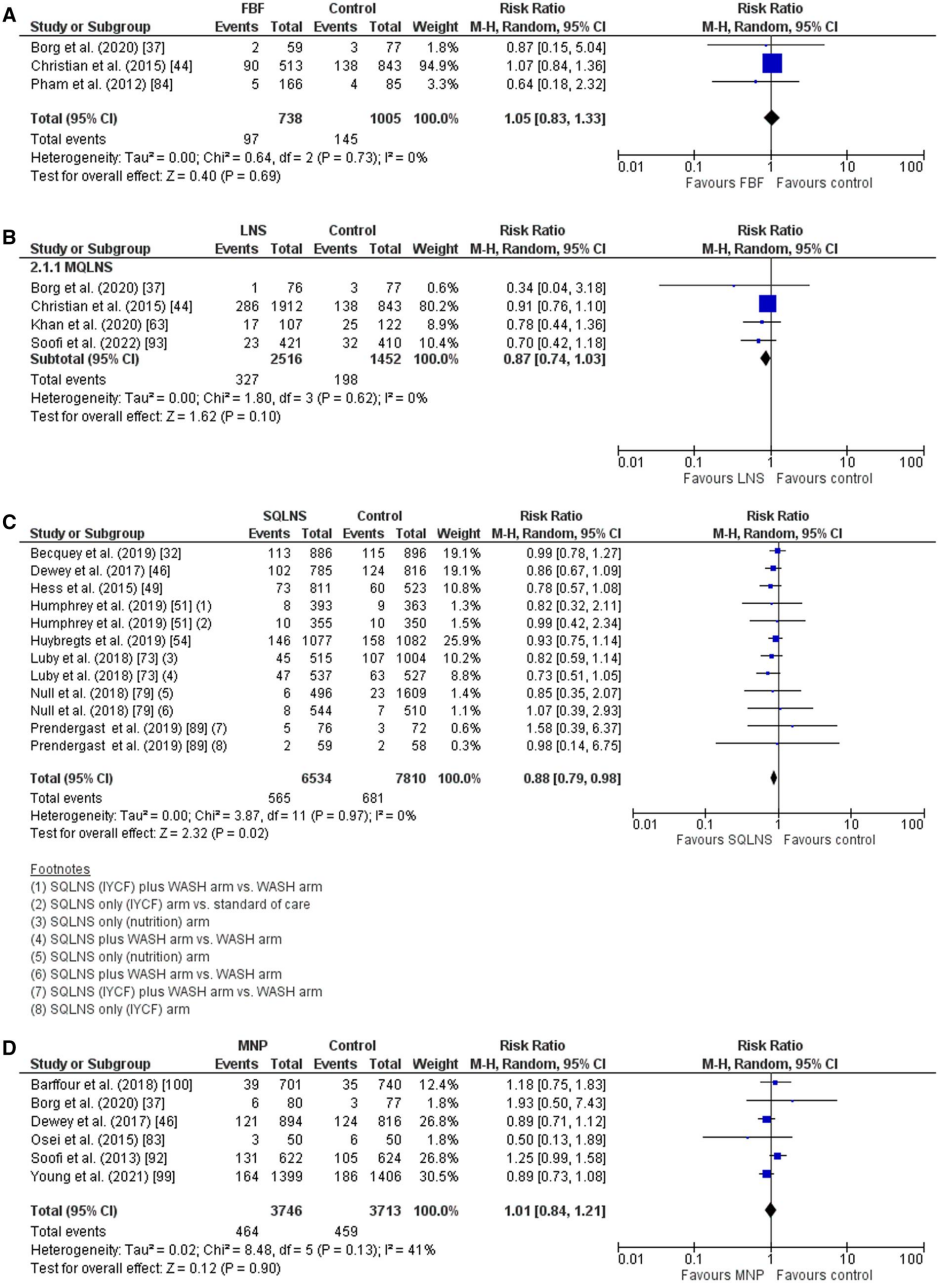
Prevalence of wasting on child supplementation. (A) FBF supplementation; (B) MQ-LNS and LQ-LNS supplementation; (C) SQ-LNS supplementation; (D) MNP supplementation. FBF indicates fortified blended food; LLQ, large quantity; LNS, lipid-based supplementation; MNP, multiple micronutrient powder; MQ, medium quantity; SQ, small quantity.

*Wasting (WHZ* *<* *−2):* Compared to the control group FBF supplementation to infants/children may have had little or no impact on the prevalence of wasting (RR, 1.05; 95% CI, 0.83-1.33; 3 trials; *I*^2^ 0%; GRADE: Low; Figure 3A),^37^^,^^44^^,^^84^ severe wasting (RR, 1.53; 95% CI, 0.26-9.07; 1 trial; GRADE: Low), and cumulative incidence of severe wasting (RR, 1.50; 95% CI, 0.25-8.88; 1 trial; GRADE: Low). None of the included trials were pooled for the incidence of wasting and for deterioration for severe wasting. Pham et al. (2012)^84^ reported on the incidence of wasting; however, due to the very low number of infants with wasting in this study statistical analysis could not be conducted.

*Anthropometric outcomes:* Supplementation of FBF given to infants/children is likely to make little or no difference in WHZ (MD, 0.02; 95% CI, −0.02 to 0.05; 4 trials; *I*^2^ 0%; GRADE: Moderate) and may have little or no impact on MUAC (0.07 cm; 95% CI, −0.05 to 0.19; 2 trials; *I*^2^ 15%; GRADE: Low), WAZ (MD, 0.07; 95% CI, −0.04 to 0.18; 4 trials; *I*^2^ 54%; GRADE: Low), and on the prevalence of underweight (RR, 0.97; 95% CI, 0.71-1.32; 3 trials; *I*^2^ 25%; GRADE: Low).

*Morbidity and mortality:* Supplementation of FBF provided to infants/children is likely to result in little or no difference in mortality (RR, 0.97; 95% CI, 0.47-2.00; 2 trials; *I*^2^ 0%; GRADE: Moderate). Such supplementation may have little or no impact on the incidence of diarrhea (RR, 0.97; 95% CI, 0.83-1.13; 1 trial; GRADE: Low), and pneumonia (RR, 0.89; 95% CI 0.69-1.14; 1 trial; GRADE: Low).

*LQ-LNS and MQ-LNS infant/child supplementation:* Eight trials^37^^,^^44^^,^^53^^,^^55^^,^^63^^,^^74^^,^^75^^,^^93^ reported on LQ-LNS and MQ-LNS supplementation given to children. Among the included studies, 1 study provided a locally produced fish-based ready-to-use supplementary food (RUSF) for 6 months^37^; 1 study provided milk-LNS and soy-LNS (MQ-LNS) for 12 months^75^; 1 study provided no-milk LNS and milk-LNS (MQ-LNS) for 12 months^74^; 1 study provided Plumpy’doz (MQ-LNS), a ready-to-eat, lipid-based nutrient supplement, for 4 months^53^; 1 study provided locally produced MQ-LNS for at least 6 months^63^; and 1 study provided ready-to-use therapeutic food (RUTF) (LQ-LNS) for 3 months.^55^ Christian et al. (2015)^44^ offered 3 types of LNS (as 3 study arms) which included Plumpy'doz, chickpea-based read-to-use food, and rice-lentil–based ready-to-use food for 12 months. Soofi et al. (2022)^93^ provided an MQ-LNS called Wawamum for 18 months. The duration of intervention ranged from 3 to 18 months. Details on the energy and quantity of LQ-LNQ and MQ-LNS supplementation are given in Table 2.^37^^,^^44^^,^^53^^,^^55^^,^^63^^,^^74^^,^^75^^,^^93^ The full evidence profiles can be found in File S6.

*Wasting (WHZ* *<* *−2):* Compared to controls, infants/children who received LQ-LNS and MQ-LNS supplementation were more likely to have a 26% reduction in the incidence of wasting (RR, 0.74; 95% CI, 0.55-0.99; 2 trials; *I*^2^ 44.5%; GRADE: Moderate) and a 45% reduction in the cumulative incidence of severe wasting (RR, 0.55; 95% CI, 0.32-0.92; 3 trials; *I*^2^ 23.2%; GRADE: Moderate). There was likely to be little or no difference in the prevalence of wasting (RR, 0.87; 95% CI, 0.74-1.03; 4 trials: *I*^2^ 0%; GRADE: Moderate; Figure 3B)^37^^,^^44^^,^^63^^,^^93^ or in the prevalence of severe wasting (RR, 0.73; 95% CI 0.12-4.32; 1 trial; GRADE: Moderate). Supplementation with LQ-LNS and MQ-LNS may have little or no impact on the cumulative incidence of wasting (RR, 0.77; 95% CI, 0.53-1.12; 3 trials; *I*^2^ 0%; GRADE: Low). None of the included trials reported deterioration to severe wasting.

*Anthropometric outcomes:* Supplementation with LQ-LNS and MQ-LNS in infants/children was likely to result in improvements in MUAC (MD, 0.14 cm; 95% CI, 0.08-0.2; 4 trials; *I*^2^ 0%; GRADE: Moderate) and WAZ (MD, 0.03; 95% CI, 0.00-0.06; 5 trials; *I*^2^ 0%; GRADE: Moderate) and reduction in the prevalence of underweight by 9% (RR, 0.91; 95% CI, 0.83-1.00; 4 trials; *I*^2^ 0%; GRADE: Moderate). However, LQ-LNS and MQ-LNS supplementation was likely to make little or no difference in WHZ (MD, 0.03; 95% CI, −0.04 to 0.11; 7 trials; *I*^2^ 64.3%; GRADE: Moderate).

*Morbidity and mortality:* Supplementation with LQ-LNS and MQ-LNS was likely to reduce mortality by 40% (RR, 0.60; 95% CI, 0.37-0.98; 7 trials; *I*^2^ 76.8%; GRADE: Moderate) and likely to make little or no difference in the incidence of diarrhea (RR, 0.97; 95% CI, 0.90-1.04; 3 trials; *I*^2^ 0%; GRADE: Moderate). Supplementation with LQ-LNS and MQ-LNS may have little or no impact on the incidence of pneumonia or respiratory infections (RR, 0.92; 95% CI, 0.80-1.07; 3 trials; *I*^2^ 66.9%; GRADE: Low).

Two trials, by Huybregts et al.^53^ and Maleta et al.,^74^ reported on morbidity outcomes but were not included in the analysis. Huybregts et al. (2012)^53^ investigated the incidence of fever and diarrhea among children 6-36 months of age and reported a lower risk of self-reported fever (22.5%; 95% CI, 14.0-30.2) in the intervention group compared to the control group, after adjusting for age, sex, socioeconomic status, and morbidity status at inclusion. Huybregts et al. also reported a lower risk of self-reported diarrhea (29.3%; 95% CI, 20.5-37.2) in the intervention group compared to the control group, after adjusting for age, sex, socioeconomic status, and morbidity status at inclusion. Maleta et al. (2015)^74^ reported on the incidence of acute respiratory infection (ARI) and the prevalence of guardian-reported fever and diarrhea. The incidence rate ratio of ARI compared to the control was reported to be 1.02 (95% CI, 0.94-1.11) in the 20-g LNS/d group and 1.02 (95% CI, 0.94-1.11) in the 40-g LNS/d group. The incidence rate ratios of fever compared to the control was reported to be 0.96 (95% CI, 0.83-1.12) in the group of children who received 20 g LNS/d and 0.99 (95% CI, 0.85-1.16) in children who received 40 g LNS/d. The prevalence of guardian-reported fever (expressed as percentage of the geometric mean ± SD) was 6.8 ± 2.5 in the 20-g LNS/d group, 6.3 ± 2.8 in the 40-g LNS/d group, and 6.7 ± 2.5 in the control group. For diarrhea, the prevalence was 3.1 ± 3.1 in the 20-g LNS/d group, 2.7 ± 3.2 in the 40-g LNS/d group, and 2.8 ± 3.0 in the control group.

*SQ-LNS infant/child supplementation:* Nine trials^32^^,^^46^^,^^49^^,^^51^^,^^54^^,^^73^^,^^74^^,^^79^^,^^89^ reported on SQ-LNS supplementation provided to infants/children. In these trials 118 kcal/d SQ-LNS (20 g/d) was provided to infants at least 6 months of age. The duration of intervention ranged from 6 to 18 months. Details on the energy and quantity of SQ-LNS are given in Table 2.^32^^,^^46^^,^^49^^,^^51^^,^^54^^,^^73^^,^^74^^,^^79^^,^^89^ The full evidence profiles can be found in File S7.

*Wasting (WHZ* *<* *−2):* Compared to controls, infants/children who received SQ-LNS supplementation were likely to have a 12% reduction in the prevalence of wasting (RR, 0.88; 95% CI, 0.79-0.98; 8 trials; *I*^2^ 0%; GRADE: Moderate; Figure 3C). Supplementation with SQ-LNS resulted in little or no difference in the prevalence of moderate wasting (RR, 0.98; 95% CI, 0.82-1.18; 2 trials; *I*^2^ 0%; GRADE: Moderate), the incidence of wasting (RR, 0.76; 95% CI, 0.57-1.02; 2 trials; *I*^2^ 0%; GRADE: Moderate),^32^^,^^46^^,^^49^^,^^51^^,^^54^^,^^73^^,^^79^^,^^89^ the cumulative incidence of wasting (RR, 0.94; 95% CI, 0.58-1.52; 1 trial; GRADE: Moderate), and the prevalence of severe wasting (RR, 0.78; 95% CI, 0.45-1.33; 2 trials; *I*^2^ 9%; GRADE: Moderate). Supplementation with SQ-LNS may have little or no impact on the cumulative incidence of severe wasting (RR, 1.82; 95% CI, 0.51-6.47; 1 trial; GRADE: Low). None of the included trials reported deterioration to severe wasting.

*Anthropometric outcomes:* Infants/children who received SQ-LNS supplementation showed an improvement in WAZ (MD, 0.14; 95% CI, 0.09-0.19; 7 trials; *I*^2^ 29%; GRADE: High) and 16% reduction in the prevalence of underweight (WAZ < −2) (RR, 0.84; 95% CI, 0.78-0.90; 7 trials; *I*^2^ 0%; GRADE: High). Supplementation with SQ-LNS provided to infants/children likely results in an increase in WHZ (MD, 0.08; 95% CI, 0.03-0.12; 9 trials; *I*^2^ 44%; GRADE: Moderate), MUAC (MD, 0.13 cm; 95% CI, 0.00-0.26; 4 trials; *I*^2^ 80%; GRADE: Moderate), and MUAC *z*-score (MUACZ; MD, 0.06; 95% CI, 0-0.11; 4 trials; *I*^2^ 0%; GRADE: Moderate).

*Morbidity and mortality:* In infants/children SQ-LNS supplementation resulted in a reduction of mortality by 24% (RR, 0.76; 95% CI, 0.63-0.91; 9 trials; *I*^2^ 0%; GRADE: High). SQ-LNS supplementation is likely to have little or no difference in the prevalence of cough or respiratory infections (RR, 0.96; 95% CI, 0.28-3.31; 2 trials; *I*^2^ 0%; GRADE: Moderate) and may have little or no impact on the prevalence of diarrhea (RR, 0.94; 95% CI, 0.80-1.11; 5 trials; *I*^2^ 49%; GRADE: Low). The findings suggest an uncertain impact of SQ-LNS on the prevalence of high fever (RR, 1.04; 95% CI, 0.80-1.34; 1 trial; GRADE: Very Low) and ARI (RR, 1.12; 95% CI, 0.77-1.64; 1 trial; GRADE: Very Low). Maleta et al. (2015)^74^ reported on the incidence of ARI and on the prevalence of guardian-reported fever and diarrhea but this study was not included in the analysis. The incidence rate ratio of ARI in the 10-g LNS/d group compared to the control was reported to be 1.02 (95% CI, 0.92-1.12). The incidence rate ratio of fever in the 10-g LNS/d group compared to the control was reported to be 0.97 (95% CI, 0.81-1.16). The prevalence of guardian-reported fever (expressed as percentage geometric mean ± SD) was 6.1 ± 2.7 in the 10-g LNS/d group and 6.7 ± 2.5 in the control group. For diarrhea, the prevalence was 3.1 ± 3.4 in the 10-g LNS/d group and 2.8 ± 3.0 in the control group.

*MNP infant/child supplementation:* Six trials^37^^,^^46^^,^^83^^,^^92^^,^^99^^,^^100^ reported on MNP supplementation to infants/children. All of the trials provided MNPs to infants. The duration of intervention ranged from 6 to 18 months. The full evidence profiles can be found in File S8.

*Wasting (WHZ* *<* *−2):* Compared to controls infants/children given MNP supplementation were likely to have little or no difference in the prevalence of wasting (RR, 1.01: 95% CI, 0.84-1.21; 6 trials; *I*^2^ 41%; GRADE: Moderate; Figure 3D).^37^^,^^46^^,^^83^^,^^92^^,^^99^^,^^100^ None of the included trials reported on the incidence of wasting, prevalence or incidence of severe wasting, or deterioration to severe wasting.

*Anthropometric outcomes:* Infants/children who received MNP supplementation were likely to have little or no difference in WHZ (MD, 0.02; 95% CI, −0.04 to 0.07; 5 trials; *I*^2^ 0%; GRADE: Moderate), MUAC (MD, 0 cm; 95% CI, −0.09 to 0.09; 2 trials; *I*^2^ 0%; GRADE: Moderate), MUACZ (MD −0.05; 9% CI, −0.13 to 0.03; 1 trial; GRADE: Moderate), WAZ (MD, 0.01; 95% CI, −0.08 to 0.09; 5 trials; *I*^2^ 63%; GRADE: Moderate), or the prevalence of underweight (RR, 1.01; 95% CI, 0.94-1.08; 6 trials; *I*^2^ 0%; GRADE: Moderate).

*Morbidity and mortality*: Infants/children who received MNP supplementation were likely to show an increase in the incidence of rapid breathing or chest indrawing (RR, 1.61; 95% CI, 1.32-1.96; 1 trial; GRADE: Moderate). An increase in the incidence of diarrhea was shown in 1 trial (RR, 1.12; 95% CI, 1.00-1.25; GRADE: Low) but pooling of the data from 3 trials showed a possible decrease in the prevalence of diarrhea (RR, 0.72; 95% CI, 0.60-0.88; *I*^2^ 0%; GRADE: Low). MNP supplementation to infants/children is likely to have little or no difference on mortality (RR, 1.04; 95% CI 0.35-3.05; 3 trials; *I*^2^ 55%; GRADE: Moderate) and may have little or no impact on the incidence of fever (RR, 0.95; 95% CI, 0.73-1.23; 1 trial; GRADE: Low) or the prevalence of fever or high fever (RR, 0.92; 95% CI, 0.83-1.01; 3 trials; *I*^2^ 0%; GARDE: Low). The findings suggest an uncertain impact of MNP supplementation on the prevalence of acute lower respiratory infection (RR, 0.85; 95% CI, 0.57-1.26; 1 trial; GRADE: Very low). Barffour et al. (2019)^100^ also reported on the incidence and prevalence of diarrhea, acute lower respiratory tract infection (ALRI), and acute upper respiratory tract infection (AURI), but this study was not included in the analysis. The incidence of diarrhea in the MNP group was found to be low (ie, 0.62 episodes per 100 days at risk) with no difference from the control group. The longitudinal prevalence of diarrhea was 1.42 per 100 days in the MNP group and 1.44 per 100 days in the control group. The incidence of ALRI was 0.006 episodes per 100 days at risk in the MNP group and 0.004 episodes per 100 days at risk in the control group. The longitudinal prevalences of ALRI in the MNP and control groups were 0.015 and 0.007 days with ALRI per 100 days observed, respectively. The incidences of AURI in the MNP and control groups were reported to be 0.09 and 0.10 episodes per 100 days at risk, respectively. The longitudinal prevalences of AURI in the MNP and control groups were 0.34 and 0.41 days with AURI per 100 days observed, respectively.

#### Infant/Child Food Intervention/Supplementation in Addition to Mothers

*SQ-LNS infant/child supplementation in addition to mothers:* Three trials^23^^,^^28^^,^^46^ reported on SQ-LNS supplementation. The trials provided 118 kcal/d (20 g/d) SQ-LNS to women during pregnancy and for 6 months postpartum. Infants also received 118 kcal/d (20 g/d) SQ-LNS at 6 months of age. The duration of intervention among infants ranged from 12 to 18 months. The full evidence profiles can be found in the File S9.

**Figure 4.**
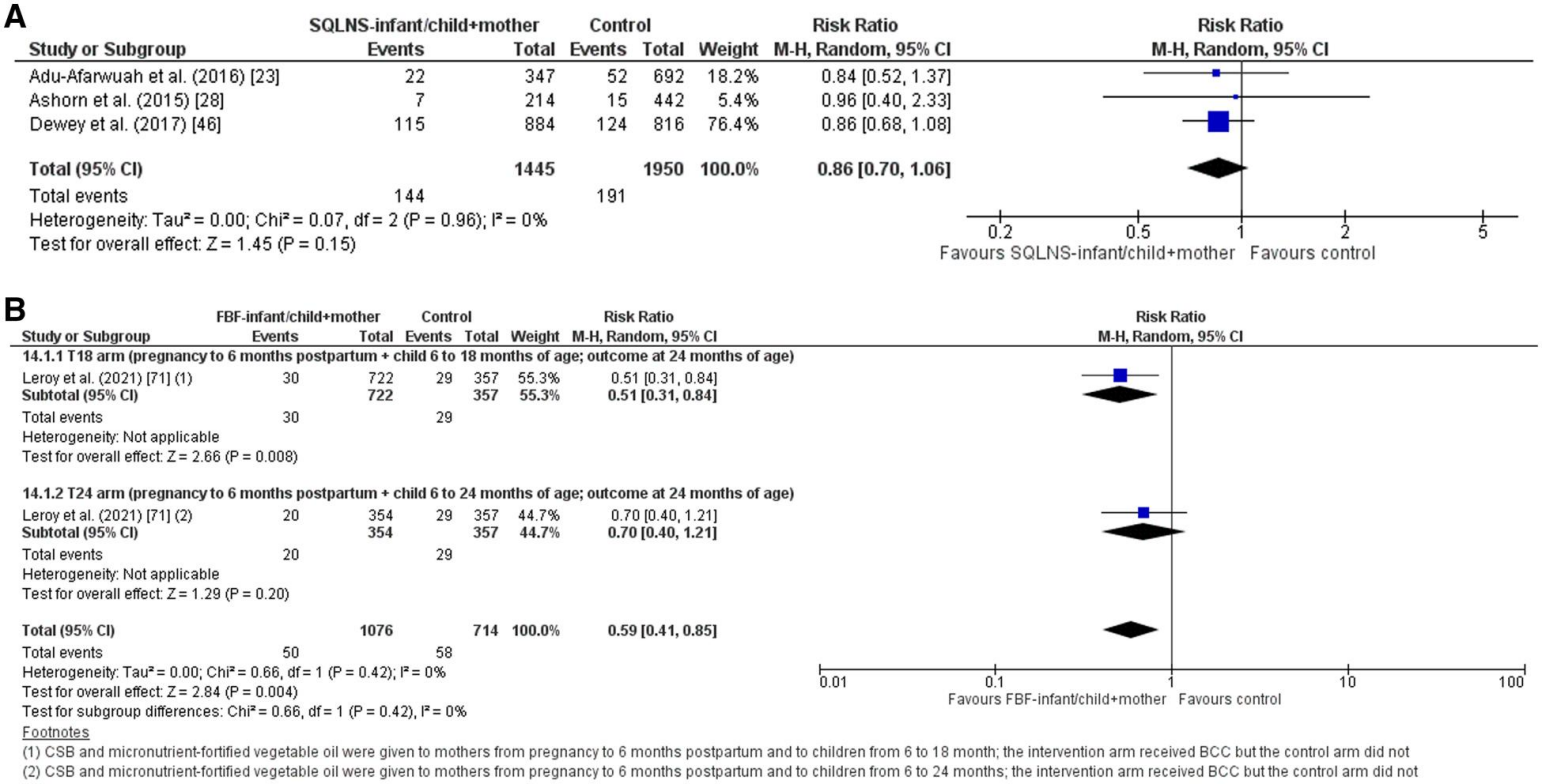
Prevalence of wasting on maternal and child supplementation. (A) SQ-LNS supplementation; (B) FBF supplementation. FBF indicates fortified blended food; LNS, lipid-based supplementation; SQ, small quantity.

*Wasting (WHZ* *<* *−2):* The results of this study suggest that SQ-LNS supplementation to infants/children and their mothers compared to controls is likely to have little or no difference in the prevalence of wasting (RR, 0.86; 95% CI, 0.70-1.06; 3 trials; *I*^2^ 0%; GRADE: Moderate; Figure 4A).^23^^,^^28^^,^^46^ In addition, SQ-LNS supplementation may have little or no impact on the prevalence of severe wasting (RR, 2.07; 95% CI, 0.42-10.15; 1 trial; GRADE: Low). None of the included trials reported the incidence of wasting and deterioration to severe wasting.

*Anthropometric outcomes:* Among the anthropometric outcomes, the reviewed study results suggest that SQ-LNS supplementation to infants/children and their mothers is likely to have little or no difference on WHZ (MD, 0.05; 95% CI, −0.03 to 0.14; 3 trials; *I*^2^ 34%; GRADE: Moderate), MUAC (MD, 0.01 cm; 95% CI, −0.18 to 0.12; 2 trials; *I*^2^ 64%; GRADE: Moderate), MUACZ (MD, 0.02; 95% CI, −0.05 to 0.09; 2 trials; *I*^2^ 0%; GRADE: Moderate), WAZ (MD, 0.10; 95% CI, −0.03 to 0.23; 3 trials; *I*^2^ 70%; GRADE: Moderate), and on the prevalence of underweight (RR, 0.93; 95% CI, 0.83-1.05; 3 trials; *I*^2^ 0%; GRADE: Moderate).

*Morbidity and mortality:* The study suggests that SQ-LNS supplementation given to infants/children and their mothers is likely to make little or no difference in the incidence of diarrhea (requiring hospitalization) (RR, 0.59; 95% CI, 0.20-1.79; 1 trial; GRADE: Moderate). In addition, SQ-LNS supplementation may have little or no impact on mortality (RR, 0.62; 95% CI, 0.24-1.58; 3 trials; *I*^2^ 39%; GRADE: Low), the prevalence of diarrhea (RR, 1.60; 95% CI 0.84-3.04; 1 trial: GRADE: Low), or the incidence of pneumonia (requiring hospitalization) (RR, 0.71; 95% CI, 0.42-1.19; 1 trial; GRADE: Low). These findings suggest an uncertain impact of SQ-LNS supplementation on the prevalence of ARI (RR, 0.96; 95% CI, 0.65-1.39; 1 trial; GRADE: Very Low) and high fever (RR, 0.83; 95% CI, 0.64-1.08; 1 trial; GRADE: Very Low).

*FBF infants/children supplementation in addition to mothers:* One trial^71^ from the African region reported on FBF supplementation to infants/children and their mothers. The trial provided corn-soy blend and micronutrient-fortified vegetable oil to mothers during pregnancy up to 6 months postpartum and children 6-24 months of age (T24 arm) or from pregnancy up to 6 months postpartum and children 6-18 months of age (T18 arm). The quantity of food supplementation is given in Table 2.^71^ The full evidence profiles can be found in File S10.

*Wasting (WHZ* *<* −*2):* The trial suggests that FBF supplementation to children up to 6-18 months of age and their mothers when compared to the control (T18 arm) is likely to reduce the prevalence of wasting by 49% (RR, 0.51; 95% CI, 0.31-0.84; 1 trial; GRADE: Moderate), while FBF supplementation to children up to 6-24 months of age in addition to their mothers (T24 arm) is likely to have little or no difference on the prevalence of wasting (RR, 0.70; 95% CI, 0.40-1.21; 1 trial; GRADE: Moderate; Figure 4B).^71^ The trial failed to report on the incidence of wasting and severe wasting, the prevalence of severe wasting, and on deterioration to severe wasting.

*Anthropometric outcomes:* Among the anthropometric outcomes, the trial suggests that FBF supplementation administered to children up to 6-18 months of age and their mothers (T18 arm) is likely to improve WHZ (MD, 0.2; 95% CI, 0.05-0.35; 1 trial; GRADE: Moderate), but FBF supplementation to mothers and children up to 6-24 months of age (T24 arm) is likely to have little or no difference on WHZ (MD 0.1; 95% CI, −0.07 to 0.27; 1 trial; GRADE: Moderate). The trial failed to report other anthropometric outcomes such as MUAC, MUACZ, WAZ, and the prevalence of underweight.

*Morbidity and mortality:* The included trial did not report on mortality and morbidity outcomes.

## DISCUSSION

In this review we aimed to assess the effectiveness of community-based food interventions/supplements for preventing wasting in children up to 5 years who are at risk of wasting and nutritional oedema. It included a total of 24 studies, comprising 19 cRCTs and 5 RCTs, conducted across the African, Southeast Asian, Eastern Mediterranean, and Western Pacific Regions. Among the included studies, 20 trials (20 articles) provided supplementation to infants/children, whereas 4 provided supplementation to infants/children in addition to mothers during pregnancy. The types of supplementation included FBFs, MQ/LQ-LNS, SQ-LNS, and multiple micronutrients.

In this review we found that infant/child supplementation with LNS provided in any quantity (ie, either SQ-LNS or MQ/LQ-LNS) or any region (ie, Southeast Asia, Africa, or Western Pacific Region) had a similar impact on wasting and anthropometric outcomes in children. The study findings suggest that SQ-LNS supplementation to infants/children significantly improved the prevalence of wasting, while supplementation with MQ/LQ-LNS to infants/children had a significant impact on reducing the incidence of wasting and cumulative incidence of severe wasting. Infant/child supplementation with both MQ/LQ-LNS and SQ-LNS showed a significant improvement in WAZ and MUAC and reducing the prevalence of underweight. Both SQ-LNS and MQ/LQ-LNS were also associated with a significant reduction in mortality rates. Infant/child supplementation with MNP was found to increase the incidence of rapid breathing/chest indrawing and diarrhea morbidity; however, the evidence for this finding is from a single study. Providing FBF supplementation to children up to 6-18 months of age in addition to mothers was found to improve WHZ and reduce the prevalence of wasting by 49%. While the majority of the included trials raised concerns regarding methodological quality based on wasting and anthropometric outcomes. On GRADE analysis, the majority of the outcomes ranged from low to moderate certainty mainly due to concerns related to overall high risk of bias and imprecision in included studies. Most of the included studies had an overall high risk of bias due to bias in the measurement of outcomes. Studies were predominantly rated as having serious imprecision when the 95% CI crossed the null threshold while simultaneously encompassing both potential benefits and harms, making it impossible to determine the true direction of effect. In some cases, when CIs didn't cross the null value, serious imprecision was noted if the intervals spanned a wide range of effect sizes (from trivial to large), creating uncertainty about the actual magnitude of benefit or harm in the population.

Wasting among infants/children can be prevented through various nutritional interventions. Similar to the findings of this review, a recent UNICEF report focusing on children in Africa highlighted the central role of LNS in wasting prevention.^114^ LNS comes in 3 main forms, ie, LQ-LNS/RUTF primarily used for severe acute malnutrition treatment, MQ-LNS/RUSF for moderate wasting treatment and prevention, and SQ-LNS.^114^ The use of LQ-LNS/RUTF for prevention purposes has been reported to be controversial, while trials on MQ-LNS and LQ-LNS have shown reductions in wasting and severe wasting among children^114^^,^^115^ While studies comparing RUSF with CSB have shown mixed results,^114^ with 1 trial reporting no difference between RUSF and CSB^116^ and a review with limited evidence suggesting LNS to be more effective than FBF (eg, CSB).^11^ The findings of this review are also consistent with those of a recently published systematic review on MQ-LNS supplementation in infants/children, which demonstrated a significant impact on reducing wasting by 11% and underweight by 6%.^117^

Despite its lower caloric content, SQ-LNS has proven effective in reducing wasting prevalence but the higher cost and sustainability of SQ-LNS remain a concern.^114^ The findings from the recent Lancet Optimising Child and Adolescent Health and Development Series^10^ suggested that SQ-LNS supplementation among children aged 6-23 months can effectively reduce moderate wasting by 17% in LMICs. Additionally, the review indicated that these interventions are generally more effective in supplementation durations exceeding 12 months. Most of the included studies in this review provided SQ-LNS supplementation to infants/children for durations ranging from 12 to 18 months. Further evidence from a recent individual participant data meta-analysis (IPD-MA) led to findings that SQ-LNS supplementation significantly reduced the prevalence of wasting by 14%^118^ and severe wasting by 31%.^119^ The IPD-MA also reported a reduction in underweight by 13% and an improvement in MUACZ by 18%.^118^

No review was found that reported on the preventive role of FBF in the prevention of wasting among children; however, explorations of the therapeutic role of FBF supplementation and FBFs have shown no impact on recovery from wasting.^120^ Supplementation with MMN has the potential to prevent micronutrient deficiency among children and improve child growth but the impact of MMN supplementation among children and pregnant women remains understudied.^114^ A 2020 systematic review on MMN supplementation in children younger than 5 years did not report on wasting, and it did not find improvement in WAZ or WHZ with MMN supplementation.^121^ Unlike this review, the earlier review did not demonstrate any impact on the incidence of diarrhea.

In this review we employed a comprehensive search strategy across multiple databases and a grey literature search, supplemented by searching bibliographies of included studies and identifying studies through expert contacts. This approach mitigates the risk of selection bias, ensuring a thorough identification of relevant literature. Additionally, the selection criteria for studies were meticulously detailed and stringent, leaving minimal room for selection bias throughout the review process. Also, the review only included RCTs, thereby ensuring that only studies with randomized intervention assignments were considered. However, this review also had limitations, in particular concerns regarding the methodological quality of the majority of the included studies, resulting in a moderate to low GRADE certainty. Therefore, future trials must prioritize robust methodologies and high-quality study designs to ensure and improve feasibility, access, compliance, and sustainability.

## CONCLUSION AND IMPLICATIONS

The findings from this review indicate the effectiveness of SQ-LNS and MQ/LQ-LNS supplementation to infants/children in improving wasting, underweight, MUAC, and mortality and of FBF supplementation to infants/children in addition to mothers can also improve wasting among children. However, methodological limitations in the majority of studies underscore the importance of prioritizing rigorous trials, with particular attention to standardized outcome measurements and longer follow-up periods. Priority should be given to investigating the optimal timing, duration, and dosage of nutritional interventions specifically for prevention rather than treatment of wasting among infants/children. Additionally, the cost-effectiveness and sustainability of these interventions in different contexts, as well as their integration within broader nutrition-sensitive and nutrition-specific programming, should be assessed in future trials.

## Supplementary Material

nuaf041_Supplementary_Data

## References

[1] World Health Organization, United Nations Children's Fund (UNICEF) & World Bank. Levels and trends in child malnutrition: UNICEF/WHO/The World Bank Group joint child malnutrition estimates: Key findings of the 2021 edition. 2021. Accessed September 23, 2023. https://iris.who.int/handle/10665/341135

[2] World Health Organization. Malnutrition. WHO. September 24th, 2020. Accessed September 24, 2020. https://www.who.int/news-room/fact-sheets/detail/malnutrition

[3] World Health Organization. Global action plan on physical activity 2018-2030: More active people for a healthier world. WHO. September 23rd, 2020. Accessed September 23, 2020. https://www.who.int/publications/i/item/9789241514187

[4] Shen Y , Cliffer IR, Suri DJ, et al Impact of stakeholder perspectives on cost-effectiveness estimates of four specialized nutritious foods for preventing stunting and wasting in children 6–23 months in Burkina Faso. Nutr J. 2020;19:20. 32106840 10.1186/s12937-020-00535-x PMC7047349

[5] Development Initiatives, 2018. 2018 Global Nutrition Report: Shining a light to spur action on nutrition. Accessed September 23, 2022. https://globalnutritionreport.org/reports/global-nutrition-report-2018/

[6] Picot J , Hartwell D, Harris P, Mendes D, Clegg A, Takeda A. The effectiveness of interventions to treat severe acute malnutrition in young children: a systematic review. Health Technol Assess. 2012;16:1-316. 10.3310/hta16190 PMC4781582 22480797

[7] Black RE , Victora CG, Walker SP, et al Maternal and Child Nutrition Study Group. Maternal and child undernutrition and overweight in low-income and middle-income countries. Lancet. 2013;382:427-451. 23746772 10.1016/S0140-6736(13)60937-X

[8] Cliffer IR , Masters WA, Perumal N, et al Monthly measurement of child lengths between 6 and 27 months of age in Burkina Faso reveals both chronic and episodic growth faltering. Am J Clin Nutr. 2022;115:94-104. 34637506 10.1093/ajcn/nqab309 PMC8755055

[9] World Health Organization. Global action plan on child wasting: A framework for action to accelerate progress in preventing and managing child wasting and the achievement of the sustainable development goals. 2020. Accessed September 22, 2022. https://www.who.int/publications/m/item/global-action-plan-on-child-wasting-a-framework-for-action

[10] Vaivada T , Lassi ZS, Irfan O, et al What can work and how? An overview of evidence-based interventions and delivery strategies to support health and human development from before conception to 20 years. Lancet. 2022;399:1810-1829. 35489360 10.1016/S0140-6736(21)02725-2

[11] Das JK , Salam RA, Hadi YB, et al Preventive lipid‐based nutrient supplements given with complementary foods to infants and young children 6 to 23 months of age for health, nutrition, and developmental outcomes. Cochrane Database Syst Rev. 2019;5:CD012611. 31046132 10.1002/14651858.CD012611.pub3 PMC6497129

[12] Shekar M , Kakietek J, Dayton Eberwein J, Walters D. An Investment Framework for Nutrition: Reaching the Global Targets for Stunting, Anemia, Breastfeeding and Wasting. World Bank; 2016.

[13] Covidence systematic review software. Veritas Health Innovation. Melbourne, Australia. Accessed July 2021. www.covidence.org

[14] Sterne J , Savović J, Page M, et al RoB 2: a revised tool for assessing risk of bias in randomised trials. BMJ. 2019;366:l4898. 31462531 10.1136/bmj.l4898

[15] Guyatt GH , Oxman AD, Vist GE, et al; GRADE Working Group. GRADE: an emerging consensus on rating quality of evidence and strength of recommendations. BMJ. 2008;336:924-926. 18436948 10.1136/bmj.39489.470347.AD PMC2335261

[16] Aakko J , Grześkowiak Ł, Asukas T, et al Lipid-based nutrient supplements do not affect gut Bifidobacterium microbiota in Malawian infants: a randomized trial. J Pediatr Gastroenterol Nutr. 2017;64:610-615. 27403608 10.1097/MPG.0000000000001333

[17] Abbeddou S , Hess SY, Yakes Jimenez E, et al Comparison of methods to assess adherence to small‐quantity lipid‐based nutrient supplements (SQ‐LNS) and dispersible tablets among young Burkinabé children participating in a community‐based intervention trial. Matern Child Nutr. 2015;11(suppl 4):90-104. 25521188 10.1111/mcn.12162 PMC6860357

[18] Abbeddou S , Jimenez EY, Hess SY, Somé JW, Ouédraogo JB, Brown KH. Small-quantity lipid-based nutrient supplements, with or without added zinc, do not cause excessive fat deposition in Burkinabe children: results from a cluster-randomized community trial. Eur J Nutr. 2022;61:4107-4120. 10.1007/s00394-022-02936-6 35829783 PMC9596589

[19] Abbeddou S , Jimenez EY, Somé JW, Ouédraogo JB, Brown KH, Hess SY. Small-quantity lipid-based nutrient supplements containing different amounts of zinc along with diarrhea and malaria treatment increase iron and vitamin A status and reduce anemia prevalence, but do not affect zinc status in young Burkinabe children: a cluster-randomized trial. BMC Pediatrics. 2017;17:46. 28152989 10.1186/s12887-016-0765-9 PMC5288861

[20] Adams KP , Adu-Afarwuah S, Mridha MK, et al The impact of maternal supplementation during pregnancy and the first 6 months postpartum on the growth status of the next child born after the intervention period: follow-up results from Bangladesh and Ghana. Matern Child Nutr. 2020;16:e12927. 10.1111/mcn.12927 32026568 PMC7083484

[21] Adams KP , Ayifah E, Phiri TE, et al Maternal and child supplementation with lipid-based nutrient supplements, but not child supplementation alone, decreases self-reported household food insecurity in some settings. J Nutr. 2017;147:2309-2318. 28978680 10.3945/jn.117.257386 PMC5697970

[22] Adu-Afarwuah S , Arnold CD, Maleta K, et al Consumption of multiple micronutrients or small-quantity lipid-based nutrient supplements containing iodine at the recommended dose during pregnancy, compared with iron and folic acid, does not affect women’s urinary iodine concentration in rural Malawi: a secondary outcome analysis of the iLiNS DYAD trial. Public Health Nutr. 2021;24:3049-3057. 33054890 10.1017/S1368980020003250 PMC9884741

[23] Adu-Afarwuah S , Lartey A, Okronipa H, et al Small-quantity, lipid-based nutrient supplements provided to women during pregnancy and 6 mo postpartum and to their infants from 6 mo of age increase the mean attained length of 18-mo-old children in semi-urban Ghana: a randomized controlled trial. Am J Clin Nutr. 2016;104:797-808. 27534634 10.3945/ajcn.116.134692 PMC4997301

[24] Adu-Afarwuah S , Lartey A, Okronipa H, et al Lipid-based nutrient supplement increases the birth size of infants of primiparous women in Ghana. Am J Clin Nutr. 2015;101:835-846. 25833980 10.3945/ajcn.114.091546

[25] Adu‐Afarwuah S , Young RT, Lartey A, et al Supplementation during pregnancy with small‐quantity lipid‐based nutrient supplements or multiple micronutrients, compared with iron and folic acid, increases women's urinary iodine concentration in semiurban Ghana: a randomized controlled trial. Matern Child Nutr. 2018;14:e12570. 29210520 10.1111/mcn.12570 PMC5900724

[26] Ahmed T , Islam M, Choudhury N, et al Results with complementary food using local food ingredients. In: Complementary Feeding: Building the Foundations for a Healthy Life. Karger Publishers; 2017:103-113. 10.1159/000448960 28315891

[27] Arnold BF , Null C, Luby SP, et al Cluster-randomised controlled trials of individual and combined water, sanitation, hygiene and nutritional interventions in rural Bangladesh and Kenya: the WASH Benefits study design and rationale. BMJ Open. 2013;3:e003476. 10.1136/bmjopen-2013-003476 PMC3758977 23996605

[28] Ashorn P , Alho L, Ashorn U, et al Supplementation of maternal diets during pregnancy and for 6 months postpartum and infant diets thereafter with small-quantity lipid-based nutrient supplements does not promote child growth by 18 months of age in rural Malawi: a randomized controlled trial. J Nutr. 2015;145:1345-1353. 25926413 10.3945/jn.114.207225

[29] Ashorn P , Alho L, Ashorn U, et al The impact of lipid-based nutrient supplement provision to pregnant women on newborn size in rural Malawi: a randomized controlled trial. Am J Clin Nutr. 2015;101:387-397. 25646337 10.3945/ajcn.114.088617

[30] Barua P , Beeson JG, Maleta K, Ashorn P, Rogerson SJ. The impact of early life exposure to Plasmodium falciparum on the development of naturally acquired immunity to malaria in young Malawian children. Malar J. 2019;18:11. 10.1186/s12936-019-2647-8 30658632 PMC6339377

[31] Barua P , Chandrasiri UP, Beeson JG, et al Effect of nutrient supplementation on the acquisition of humoral immunity to Plasmodium falciparum in young Malawian children. Malaria J. 2018;17:74. 10.1186/s12936-018-2224-6 PMC5804088 29415730

[32] Becquey E , Huybregts L, Zongrone A, et al Impact on child acute malnutrition of integrating a preventive nutrition package into facility-based screening for acute malnutrition during well-baby consultation: a cluster-randomized controlled trial in Burkina Faso. PLoS Med. 2019;16:e1002877. 31454347 10.1371/journal.pmed.1002877 PMC6711504

[33] Bendabenda J , Alho L, Ashorn U, et al The effect of providing lipid-based nutrient supplements on morbidity in rural Malawian infants and young children: a randomized controlled trial. Public Health Nutr. 2016;19:1893-1903. 26956611 10.1017/S1368980016000331 PMC10271160

[34] Bendabenda J , Patson N, Hallamaa L, et al Does anthropometric status at 6 months predict the over-dispersion of malaria infections in children aged 6–18 months? A prospective cohort study. Malar J. 2019;18:143. 31010435 10.1186/s12936-019-2778-y PMC6477714

[35] Bendabenda J , Patson N, Hallamaa L, et al The association of malaria morbidity with linear growth, hemoglobin, iron status, and development in young Malawian children: a prospective cohort study. BMC Pediatr. 2018;18:396. 30593271 10.1186/s12887-018-1378-2 PMC6309082

[36] Borg B , Mihrshahi S, Griffin M, Chamnan C, Laillou A, Wieringa FT. Crossover trial to test the acceptability of a locally produced lipid-based nutrient supplement (LNS) for children under 2 years in Cambodia: a study protocol. BMJ Open. 2017;7:e015958. 10.1136/bmjopen-2017-015958 PMC5588973 28882910

[37] Borg B , Sok D, Mihrshahi S, et al Effectiveness of a locally produced ready-to-use supplementary food in preventing growth faltering for children under 2 years in Cambodia: a cluster randomised controlled trial. Matern Child Nutr. 2020;16:e12896. 10.1111/mcn.12896 31885221 PMC7038903

[38] Campbell RK , Hurley KM, Shamim AA, et al Effect of complementary food supplementation on breastfeeding and home diet in rural Bangladeshi children. Am J Clin Nutr. 2016;104:1450-1458. 27680994 10.3945/ajcn.116.135509 PMC5081719

[39] Campbell RK , Hurley KM, Shamim AA, et al Complementary food supplements increase dietary nutrient adequacy and do not replace home food consumption in children 6–18 months old in a randomized controlled trial in rural Bangladesh. J Nutr. 2018;148:1484-1492. 30184222 10.1093/jn/nxy136

[40] Campbell RK , Schulze K, Shaikh S, et al Biomarkers of environmental enteric dysfunction among children in rural Bangladesh. J Pediatr Gastroenterol Nutr. 2017;65:40-46. 28644348 10.1097/MPG.0000000000001557 PMC5492885

[41] Chandrasiri UP , Fowkes FJ, Beeson JG, et al Association between malaria immunity and pregnancy outcomes among Malawian pregnant women receiving nutrient supplementation. Malar J. 2016;15:547-549. 27829430 10.1186/s12936-016-1597-7 PMC5103486

[42] Chandrasiri UP , Fowkes FJ, Richards JS, et al The impact of lipid-based nutrient supplementation on anti-malarial antibodies in pregnant women in a randomized controlled trial. Malar J. 2015;14:193-112. 25957793 10.1186/s12936-015-0707-2 PMC4438573

[43] Chowdhury ZT , Hurley KM, Campbell RK, et al Novel method for estimating nutrient intakes using a semistructured 24-hour diet recall for infants and young children in rural Bangladesh. Curr Dev Nutr. 2020;4:nzaa123. 32875267 10.1093/cdn/nzaa123 PMC7447588

[44] Christian P , Shaikh S, Shamim AA, et al Effect of fortified complementary food supplementation on child growth in rural Bangladesh—a cluster-randomized trial. Int J Epidemiol. 2015;44:1862-1876. doi: 10.1093/ije/dyv155. Epub 2015 Aug 14. 26275453 PMC4689999

[45] Dewey KG , Matias SL, Mridha MK, Arnold CD. Nutrient supplementation during the first 1000 days and growth of infants born to pregnant adolescents. Ann N Y Acad Sci. 2020;1468:25-34. 31378980 10.1111/nyas.14191 PMC7317730

[46] Dewey KG , Mridha MK, Matias SL, et al Lipid-based nutrient supplementation in the first 1000 d improves child growth in Bangladesh: a cluster-randomized effectiveness trial. Am J Clin Nutr. 2017;105:944-957. 10.3945/ajcn.116.147942 28275125

[47] Doyle R , Gondwe A, Fan Y-M, et al A Lactobacillus-deficient vaginal microbiota dominates postpartum women in rural Malawi. Appl Environ Microbiol. 2018;84:e02150-17. 29305501 10.1128/AEM.02150-17 PMC5835753

[48] Hess SY , Abbeddou S, Jimenez EY, Ouédraogo J-B, Brown KH. Iodine status of young Burkinabe children receiving small-quantity lipid-based nutrient supplements and iodised salt: a cluster-randomised trial. Br J Nutr. 2015;114:1829-1837. 26411504 10.1017/S0007114515003554

[49] Hess SY , Abbeddou S, Jimenez EY, et al Small-quantity lipid-based nutrient supplements, regardless of their zinc content, increase growth and reduce the prevalence of stunting and wasting in young Burkinabe children: a cluster-randomized trial. PloS One. 2015;10:e0122242. 10.1371/journal.pone.0122242 25816354 PMC4376671

[50] Hess SY , Peerson JM, Becquey E, et al Differing growth responses to nutritional supplements in neighboring health districts of Burkina Faso are likely due to benefits of small-quantity lipid-based nutrient supplements (LNS). PLoS One. 2017;12:e0181770. 28771493 10.1371/journal.pone.0181770 PMC5542440

[51] Humphrey JH , Mbuya MN, Ntozini R, et al Sanitation Hygiene Infant Nutrition Efficacy (SHINE) Trial Team. Independent and combined effects of improved water, sanitation, and hygiene, and improved complementary feeding, on child stunting and anaemia in rural Zimbabwe: a cluster-randomised trial. Lancet Global Health. 2019;7:e132-e147. 30554749 10.1016/S2214-109X(18)30374-7 PMC6293965

[52] Huybregts L , Becquey E, Zongrone A, et al The impact of integrated prevention and treatment on child malnutrition and health: the PROMIS project, a randomized control trial in Burkina Faso and Mali. BMC Public Health. 2017;17:237-211. 28274214 10.1186/s12889-017-4146-6 PMC5343313

[53] Huybregts L , Houngbé F, Salpéteur C, et al The effect of adding ready-to-use supplementary food to a general food distribution on child nutritional status and morbidity: a cluster-randomized controlled trial. PLoS Medicine. 2012;9:e1001313. 10.1371/journal.pmed.1001313 23028263 PMC3445445

[54] Huybregts L , Le Port A, Becquey E, et al Impact on child acute malnutrition of integrating small-quantity lipid-based nutrient supplements into community-level screening for acute malnutrition: a cluster-randomized controlled trial in Mali. PLoS Med. 2019;16:e1002892. 10.1371/journal.pmed.1002892 31454356 PMC6711497

[55] Isanaka S , Nombela N, Djibo A, et al Effect of preventive supplementation with ready-to-use therapeutic food on the nutritional status, mortality, and morbidity of children aged 6 to 60 months in Niger: a cluster randomized trial. JAMA. 2009;301:277-285. 19155454 10.1001/jama.2008.1018 PMC3144630

[56] Isanaka S , Roederer T, Djibo A, et al Reducing wasting in young children with preventive supplementation: a cohort study in Niger. Pediatrics. 2010;126:e442-e450. 20660552 10.1542/peds.2009-2814 PMC3144628

[57] Jorgensen JM , Arnold C, Ashorn P, et al Lipid-based nutrient supplements during pregnancy and lactation did not affect human milk oligosaccharides and bioactive proteins in a randomized trial. J Nutr. 2017;147:1867-1874. 28794206 10.3945/jn.117.252981 PMC5610548

[58] Jorgensen JM , Young R, Ashorn P, et al Associations of human milk oligosaccharides and bioactive proteins with infant growth and development among Malawian mother-infant dyads. Am J Clin Nutr. 2021;113:209-220. 33096556 10.1093/ajcn/nqaa272 PMC7779225

[59] Jorgensen JM , Young R, Ashorn P, et al Associations of human milk oligosaccharides and bioactive proteins with infant morbidity and inflammation in Malawian mother-infant dyads. Curr Dev Nutr. 2021;5:nzab072. 34084993 10.1093/cdn/nzab072 PMC8163417

[60] Kamng'ona AW , Young R, Arnold CD, et al Provision of lipid-based nutrient supplements to mothers during pregnancy and 6 months postpartum and to their infants from 6 to 18 months promotes infant gut microbiota diversity at 18 months of age but not microbiota maturation in a rural Malawian setting: secondary outcomes of a randomized trial. J Nutr. 2020;150:918-928. 31909811 10.1093/jn/nxz298 PMC7138685

[61] Keats EC , Das JK, Bhutta ZA. Micronutrient powders and diarrhoea risk in infants and young children—Authors' reply. Lancet Child Adolesc Health. 2021;5:e29-e30. 34302748 10.1016/S2352-4642(21)00164-4

[62] Khan GN , Kureishy S, Ariff S, et al Specialized nutritious food combined with cash transfers and social and behavior change communication to prevent stunting among children aged 6 to 23 months in Pakistan: protocol for a cluster randomized controlled trial. JMIR Res Protoc. 2020;9:e19001. 32831183 10.2196/19001 PMC7477667

[63] Khan GN , Kureishy S, Ariff S, et al Effect of lipid-based nutrient supplement-medium quantity on reduction of stunting in children 6-23 months of age in Sindh, Pakistan: a cluster randomized controlled trial. PloS One. 2020;15:e0237210. 10.1371/journal.pone.0237210 32790725 PMC7425934

[64] Klevor MK , Adu-Afarwuah S, Ashorn P, et al A mixed method study exploring adherence to and acceptability of small quantity lipid-based nutrient supplements (SQ-LNS) among pregnant and lactating women in Ghana and Malawi. BMC Pregnancy Childbirth. 2016;16:253. 27577112 10.1186/s12884-016-1039-0 PMC5004276

[65] Kortekangas E , Young R, Cheung YB, et al A prospective study on child morbidity and gut microbiota in rural Malawi. J Pediatr Gastroenterol Nutr. 2019;69:431-437. 31436705 10.1097/MPG.0000000000002435

[66] Kumwenda C , Dewey KG, Hemsworth J, Ashorn P, Maleta K, Haskell MJ. Lipid-based nutrient supplements do not decrease breast milk intake of Malawian infants. Am J Clin Nutr. 2014;99:617-623. 24368436 10.3945/ajcn.113.076588

[67] Larson LM , Young MF, Bauer PJ, et al Effectiveness of a home fortification programme with multiple micronutrients on infant and young child development: a cluster-randomised trial in rural Bihar, India. Br J Nutr. 2018;120:176-187. 29947323 10.1017/S000711451800140X PMC6088539

[68] Larson LM , Young MF, Ramakrishnan U, et al A cross-sectional survey in rural Bihar, India, indicates that nutritional status, diet, and stimulation are associated with motor and mental development in young children. J Nutr. 2017;147:1578-1585. 28615374 10.3945/jn.117.251231 PMC5525111

[69] Leroy JL , K Olney D, Bliznashka L, Ruel M. Tubaramure, a food-assisted maternal and child health and nutrition program in Burundi, increased household food security and energy and micronutrient consumption, and maternal and child dietary diversity: a cluster-randomized controlled trial. J Nutr. 2020;150:945-957. 31858128 10.1093/jn/nxz295 PMC7138675

[70] Leroy JL , Olney D, Ruel M. Tubaramure, a food-assisted integrated health and nutrition program, reduces child stunting in Burundi: a cluster-randomized controlled intervention trial. J Nutr. 2018;148:445-452. 29546306 10.1093/jn/nxx063

[71] Leroy JL , Olney DK, Nduwabike N, Ruel MT. Tubaramure, a food-assisted integrated health and nutrition program, reduces child wasting in Burundi: a cluster-randomized controlled intervention trial. J Nutr. 2021;151:197-205. 33245129 10.1093/jn/nxaa330 PMC7717329

[72] Leroy JL , Olney DK, Ruel MT. PROCOMIDA, a food-assisted maternal and child health and nutrition program, contributes to postpartum weight retention in Guatemala: a cluster-randomized controlled intervention trial. J Nutr. 2019;149:2219-2227. 31373374 10.1093/jn/nxz175 PMC6888017

[73] Luby SP , Rahman M, Arnold BF, et al Effects of water quality, sanitation, handwashing, and nutritional interventions on diarrhoea and child growth in rural Bangladesh: a cluster randomised controlled trial. Lancet Glob Health. 2018;6:e302-e315. 29396217 10.1016/S2214-109X(17)30490-4 PMC5809718

[74] Maleta KM , Phuka J, Alho L, et al Provision of 10-40 g/d lipid-based nutrient supplements from 6 to 18 months of age does not prevent linear growth faltering in Malawi. J Nutr. 2015;145:1909-1915. 10.3945/jn.114.208181 26063066

[75] Mangani C , Maleta K, Phuka J, et al Effect of complementary feeding with lipid-based nutrient supplements and corn-soy blend on the incidence of stunting and linear growth among 6- to 18-month-old infants and children in rural Malawi. Matern Child Nutr. 2015;11(suppl 4):132-143. 10.1111/mcn.12068 23795976 PMC6860208

[76] Matias SL , Mridha MK, Young RT, et al Prenatal and postnatal supplementation with lipid-based nutrient supplements reduces anemia and iron deficiency in 18-month-old Bangladeshi children: a cluster-randomized effectiveness trial. J Nutr. 2018;148:1167-1176. 29901736 10.1093/jn/nxy078

[77] Mridha MK , Matias SL, Chaparro CM, et al Lipid-based nutrient supplements for pregnant women reduce newborn stunting in a cluster-randomized controlled effectiveness trial in Bangladesh. Am J Clin Nutr. 2016;103:236-249. 10.3945/ajcn.115.111336 26607935 PMC6443293

[78] Nkhoma M , Ashorn P, Ashorn U, et al Providing lipid-based nutrient supplement during pregnancy does not reduce the risk of maternal P falciparum parasitaemia and reproductive tract infections: a randomised controlled trial. BMC Pregnancy Childbirth. 2017;17:35-38. 28095801 10.1186/s12884-016-1215-2 PMC5240436

[79] Null C , Stewart CP, Pickering AJ, et al Effects of water quality, sanitation, handwashing, and nutritional interventions on diarrhoea and child growth in rural Kenya: a cluster-randomised controlled trial. Lancet Glob Health. 2018;6:e316-e329. 10.1016/S2214-109X(18)30005-6 29396219 PMC5809717

[80] Oaks BM , Jorgensen JM, Baldiviez LM, et al Prenatal iron deficiency and replete iron status are associated with adverse birth outcomes, but associations differ in Ghana and Malawi. J Nutr. 2019;149:513-521. 30629202 10.1093/jn/nxy278 PMC6398386

[81] Oaks BM , Young RR, Adu-Afarwuah S, et al Effects of a lipid-based nutrient supplement during pregnancy and lactation on maternal plasma fatty acid status and lipid profile: results of two randomized controlled trials. Prostaglandins Leukot Essent Fatty Acids. 2017;117:28-35. 28237085 10.1016/j.plefa.2017.01.007 PMC5338685

[82] Olney DK , Leroy J, Bliznashka L, Ruel MT. PROCOMIDA, a food-assisted maternal and child health and nutrition program, reduces child stunting in Guatemala: a cluster-randomized controlled intervention trial. J Nutr. 2018;148:1493-1505. 30184223 10.1093/jn/nxy138 PMC6118165

[83] Osei AK , Pandey P, Spiro D, et al Adding multiple micronutrient powders to a homestead food production programme yields marginally significant benefit on anaemia reduction among young children in Nepal. Matern Child Nutr. 2015;11(suppl 4):188-202. 25682798 10.1111/mcn.12173 PMC6860240

[84] Pham P V , Hoan NV, Salvignol B, et al A six-month intervention with two different types of micronutrient-fortified complementary foods had distinct short- and long-term effects on linear and Ponderal growth of Vietnamese infants. J Nutr. 2012;142:1735-1740. 10.3945/jn.111.154211 22810985

[85] Prado EL , Abbeddou S, Adu-Afarwuah S, et al Linear growth and child development in Burkina Faso, Ghana, and Malawi. Pediatrics. 2016;138:10.1542/peds.2015-4698 27474016

[86] Prado EL , Abbeddou S, Adu‐Afarwuah S, et al Predictors and pathways of language and motor development in four prospective cohorts of young children in Ghana, Malawi, and Burkina Faso. J Child Psychol Psychiatry. 2017;58:1264-1275. 28543426 10.1111/jcpp.12751 PMC5697619

[87] Prado EL , Abbeddou S, Yakes Jimenez E, et al Lipid-based nutrient supplements plus malaria and diarrhea treatment increase infant development scores in a cluster-randomized trial in Burkina Faso. J Nutr. 2015;146:814-822. 26962193 10.3945/jn.115.225524

[88] Prado EL , Maleta K, Ashorn P, et al Effects of maternal and child lipid-based nutrient supplements on infant development: a randomized trial in Malawi. Am J Clin Nutr. 2016;103:784-793. 26843155 10.3945/ajcn.115.114579

[89] Prendergast AJ , Chasekwa B, Evans C, et al; SHINE Trial Team. Independent and combined effects of improved water, sanitation, and hygiene, and improved complementary feeding, on stunting and anaemia among HIV-exposed children in rural Zimbabwe: a cluster-randomised controlled trial. Lancet Child Adolesc Health. 2019;3:77-90. 30573417 10.1016/S2352-4642(18)30340-7 PMC6472652

[90] Shaikh S , Campbell RK, Mehra S, et al Supplementation with fortified lipid-based and blended complementary foods has variable impact on body composition among rural Bangladeshi children: a cluster-randomized controlled trial. J Nutr. 2020;150:1924-1932. 32240304 10.1093/jn/nxaa061 PMC7330466

[91] Somé JW , Abbeddou S, Jimenez EY, et al Effect of zinc added to a daily small-quantity lipid-based nutrient supplement on diarrhoea, malaria, fever and respiratory infections in young children in rural Burkina Faso: a cluster-randomised trial. BMJ Open. 2015;5:e007828. 10.1136/bmjopen-2015-007828 PMC4567679 26362661

[92] Soofi S , Cousens S, Iqbal SP, et al Effect of provision of daily zinc and iron with several micronutrients on growth and morbidity among young children in Pakistan: a cluster-randomised trial. Lancet. 2013;382:29-40. 10.1016/s0140-6736(13)60437-7 23602230

[93] Soofi SB , Ariff S, Khan GN, et al Effectiveness of unconditional cash transfers combined with lipid-based nutrient supplement and/or behavior change communication to prevent stunting among children in Pakistan: a cluster randomized controlled trial. Am J Clin Nutr. 2022;115:492-502. 34612491 10.1093/ajcn/nqab341 PMC8827069

[94] Stewart CP , Oaks BM, Laugero KD, et al Maternal cortisol and stress are associated with birth outcomes, but are not affected by lipid-based nutrient supplements during pregnancy: an analysis of data from a randomized controlled trial in rural Malawi. BMC Pregnancy Childbirth. 2015;15:346. 26694646 10.1186/s12884-015-0793-8 PMC4688934

[95] Humphrey JH , Jones AD, Manges A, et al Team SHINET. The sanitation hygiene infant nutrition efficacy (SHINE) trial: rationale, design, and methods. Clin Infect Dis. 2015;61(suppl 7):S685-S702. 26602296 10.1093/cid/civ844 PMC4657589

[96] Ullah MB , Mridha MK, Arnold CD, et al Provision of pre-and postnatal nutritional supplements generally did not increase or decrease common childhood illnesses in Bangladesh: a cluster-randomized effectiveness trial. J Nutr. 2019;149:1271-1281. 31162588 10.1093/jn/nxz059

[97] Ullah MB , Mridha MK, Arnold CD, et al Newborn physical condition and breastfeeding behaviours: secondary outcomes of a cluster‐randomized trial of prenatal lipid‐based nutrient supplements in Bangladesh. Matern Child Nutr. 2019;15:e12844. 31106491 10.1111/mcn.12844 PMC6859973

[98] Van Phu P , Van Hoan N, Salvignol B, et al Complementary foods fortified with micronutrients prevent iron deficiency and anemia in Vietnamese infants. J Nutr. 2010;140:2241-2247. 20980657 10.3945/jn.110.123711

[99] Young MF , Mehta RV, Gosdin L, et al Home fortification of complementary foods reduces anemia and diarrhea among children aged 6–18 months in Bihar, India: a large-scale effectiveness trial. J Nutr. 2021;151:1983-1992. 33880566 10.1093/jn/nxab065 PMC8245869

[100] Barffour MA , Hinnouho G-M, Kounnavong S, et al Effects of daily zinc, daily multiple micronutrient powder, or therapeutic zinc supplementation for diarrhea prevention on physical growth, anemia, and micronutrient status in rural Laotian children: a randomized controlled trial. J Pediatr. 2019;207:80-89.e2. 30580974 10.1016/j.jpeds.2018.11.022 PMC6448681

[101] Barffour MA , Hinnouho G-M, Wessells KR, et al Effects of therapeutic zinc supplementation for diarrhea and two preventive zinc supplementation regimens on the incidence and duration of diarrhea and acute respiratory tract infections in rural Laotian children: a randomized controlled trial. J Global Health. 2020;10:010424. 10.7189/jogh.10.010424 PMC7321011 32612816

[102] Hess SY , Hinnouho G-M, Barffour MA, et al First field test of an innovative, wider tape to measure mid-upper arm circumference in young Laotian children. Food Nutr Bull. 2018;39:28-38. 29258337 10.1177/0379572117742502

[103] Hess SY , Wessells KR, Hinnouho G-M, et al Iron status and inherited haemoglobin disorders modify the effects of micronutrient powders on linear growth and morbidity among young Lao children in a double-blind randomised trial. Br J Nutr. 2019;122:895-909. 31303184 10.1017/S0007114519001715 PMC7672373

[104] Hinnouho G-M , Barffour MA, Wessells KR, et al Comparison of haemoglobin assessments by HemoCue and two automated haematology analysers in young Laotian children. J Clin Pathol. 2018;71:532-538. 29197856 10.1136/jclinpath-2017-204786 PMC5969348

[105] Hinnouho G-M , Bernstein RM, Barffour MA, et al Impact of two forms of daily preventive zinc or therapeutic zinc supplementation for diarrhea on hair cortisol concentrations among rural Laotian children: a randomized controlled trial. Nutrients. 2018;11:47. 30591656 10.3390/nu11010047 PMC6356851

[106] Hinnouho G-M , Hampel D, Shahab-Ferdows S, et al Daily supplementation of a multiple micronutrient powder improves folate but not thiamine, riboflavin, or vitamin B12 status among young Laotian children: a randomized controlled trial. Eur J Nutr. 2022;61:3423-3435. 35534778 10.1007/s00394-022-02890-3 PMC9464137

[107] Hinnouho G-M , Wessells KR, Barffour MA, et al Impact of different strategies for delivering supplemental zinc on selected fecal markers of environmental enteric dysfunction among young Laotian children: a randomized controlled trial. Am J Trop Med Hygiene. 2020;103:1416-1426. 10.4269/ajtmh.20-0106 PMC7543857 32618258

[108] Kewcharoenwong C , Schuster GU, Wessells KR, et al Daily preventive zinc supplementation decreases lymphocyte and eosinophil concentrations in rural Laotian children from communities with a high prevalence of zinc deficiency: results of a randomized controlled trial. J Nutr. 2020;150:2204-2213. 32119742 10.1093/jn/nxaa037

[109] Kewcharoenwong C , Sein MM, Nithichanon A, et al Daily preventive zinc supplementation increases the antibody response against pathogenic Escherichia coli in children with zinc insufficiency: a randomised controlled trial. Sci Rep. 2022;12:16084. 36167891 10.1038/s41598-022-20445-8 PMC9515173

[110] Kingchaiyaphum B , Sanchaisuriya K, Fucharoen G, et al Hemoglobins F, A2, and E levels in Laotian children aged 6‐23 months with Hb E disorders: effect of age, sex, and thalassemia types. Int J Lab Hematol. 2020;42:277-283. 32048804 10.1111/ijlh.13164 PMC7318314

[111] Wessells KR , Brown KH, Arnold CD, et al Plasma and nail zinc concentrations, but not hair zinc, respond positively to two different forms of preventive zinc supplementation in young Laotian children: a randomized controlled trial. Biol Trace Elem Res. 2021;199:442-452. 32356207 10.1007/s12011-020-02163-2 PMC7746564

[112] Wessells KR , Brown KH, Kounnavong S, et al Comparison of two forms of daily preventive zinc supplementation versus therapeutic zinc supplementation for diarrhea on young children’s physical growth and risk of infection: study design and rationale for a randomized controlled trial. BMC Nutr. 2018;4:39. 32153900 10.1186/s40795-018-0247-6 PMC7050875

[113] Wessells KR , Hinnouho G-M, Barffour MA, et al Impact of daily preventive zinc or therapeutic zinc supplementation for diarrhea on plasma biomarkers of environmental enteric dysfunction among rural Laotian children: a randomized controlled trial. Am J Trop Med Hyg. 2020;102:415-426. 31889508 10.4269/ajtmh.19-0584 PMC7008314

[114] United Nations International Children's Emergency Fund. Evidence booster on wasting prevention: risk factors, research results and integrated packages. 2020. Accessed November 21, 2024. https://www.unicef.org/wca/media/5671/file/Evidence-Booster-on-Wasting-Prevention-in-West-and-Central-Africa.pdf

[115] Neufeld LM. Ready-to-use therapeutic food for the prevention of wasting in children. JAMA. 2009;301:327-328. 19155462 10.1001/jama.2008.1023

[116] Thakwalakwa CM , Ashorn P, Jawati M, Phuka JC, Cheung YB, Maleta KM. An effectiveness trial showed lipid-based nutrient supplementation but not corn–soya blend offered a modest benefit in weight gain among 6-to 18-month-old underweight children in rural Malawi. Public Health Nutr. 2012;15:1755-1762. 22691922 10.1017/S1368980012003023

[117] Dewey KG , Arnold CD, Wessells KR, Stewart CP. Lipid-based nutrient supplements for prevention of child undernutrition: when less may be more. Am J Clin Nutr. 2023;118:1133-1144. 37742931 10.1016/j.ajcnut.2023.09.007

[118] Dewey KG , Wessells KR, Arnold CD, et al Characteristics that modify the effect of small-quantity lipid-based nutrient supplementation on child growth: an individual participant data meta-analysis of randomized controlled trials. Am J Clin Nutr. 2021;114:15S-42S. 34590672 10.1093/ajcn/nqab278 PMC8560308

[119] Dewey KG , Arnold CD, Wessells KR, et al Preventive small-quantity lipid-based nutrient supplements reduce severe wasting and severe stunting among young children: an individual participant data meta-analysis of randomized controlled trials. Am J Clin Nutr. 2022;116:1314-1333. 36045000 10.1093/ajcn/nqac232

[120] Cichon B , Das JK, Salam RA, et al Effectiveness of dietary management for moderate wasting among children > 6 months of age-a systematic review and meta-analysis exploring different types, quantities, and durations. Nutrients. 2023;15:1076. 10.3390/nu15051076 PMC10005276 36904076

[121] Tam E , Keats EC, Rind F, Das JK, Bhutta ZA. Micronutrient supplementation and fortification interventions on health and development outcomes among children under-five in low-and middle-income countries: a systematic review and meta-analysis. Nutrients. 2020;12:289. 31973225 10.3390/nu12020289 PMC7071447

