# Supplementary material for: Community-Based Child Food Interventions/Supplements for the Prevention of Wasting in Children Up to 5 Years at Risk of Wasting and Nutritional Oedema: A Systematic Review and Meta-Analysis
Source: Nutr Rev. 2025 Apr 24;83(8):1402–24. doi: 10.1093/nutrit/nuaf041 (PMC12241862; doi:10.1093/nutrit/nuaf041)
Supplement: nuaf041_Supplementary_Data [file nuaf041_supplementary_data.zip › nuaf041_Supplementary_Data/Supporting file 10.docx]

**Intervention:** FBFs - maternal & infant/child supplementation

| **Certainty assessment** | | | | | | | **№ of patients** | | **Effect** | | **Certainty** | **Importance** |
| --- | --- | --- | --- | --- | --- | --- | --- | --- | --- | --- | --- | --- |
| **№ of studies** | **Study design** | **Risk of bias** | **Inconsistency** | **Indirectness** | **Imprecision** | **Other considerations** | **FBFs - maternal & infant/child - UPDATED WITH CORRECT ARMS** | **control** | **Relative (95% CI)** | **Absolute (95% CI)** |  |  |
| **Prevalence of wasting - T18 arm (pregnancy to 6 months postpartum + child 6 to 18 months of age; outcome at 24 months of age)** | | | | | | | | | | | | |
| 1 | randomised trials | not serious | not serious | not serious | serious^a^ | none | 30/722 (4.2%) | 29/357 (8.1%) | **RR 0.51** (0.31 to 0.84) | **40 fewer per 1,000** (from 56 fewer to 13 fewer) | ⨁⨁⨁◯ Moderate | CRITICAL |
| **Prevalence of wasting - T24 arm (pregnancy to 6 months postpartum + child 6 to 24 months of age; outcome at 24 months of age)** | | | | | | | | | | | | |
| 1 | randomised trials | not serious | not serious | not serious | serious^b^ | none | 20/354 (5.6%) | 29/357 (8.1%) | **RR 0.70** (0.40 to 1.21) | **24 fewer per 1,000** (from 49 fewer to 17 more) | ⨁⨁⨁◯ Moderate | CRITICAL |
| **Deterioration to severe wasting - not measured** | | | | | | | | | | | | |
| - | - | - | - | - | - | - | - | - | - | - | - | CRITICAL |
| **WHZ - T18 arm (pregnancy to 6 months postpartum + child 6 to 18 months of age; outcome at 24 months of age)** | | | | | | | | | | | | |
| 1 | randomised trials | not serious | not serious | not serious | serious^a^ | none | 722 | 357 | - | MD **0.2 higher** (0.05 higher to 0.35 higher) | ⨁⨁⨁◯ Moderate | IMPORTANT |
| **WHZ - T24 arm (pregnancy to 6 months postpartum + child 6 to 24 months of age; outcome at 24 months of age)** | | | | | | | | | | | | |
| 1 | randomised trials | not serious | not serious | not serious | serious^b^ | none | 354 | 357 | - | MD **0.1 higher** (0.07 lower to 0.27 higher) | ⨁⨁⨁◯ Moderate | IMPORTANT |
| **Morbidity - not measured** | | | | | | | | | | | | |
| - | - | - | - | - | - | - | - | - | - | - | - | IMPORTANT |
| **Mortality - not measured** | | | | | | | | | | | | |
| - | - | - | - | - | - | - | - | - | - | - | - | IMPORTANT |

**CI:** confidence interval; **MD:** mean difference; **RR:** risk ratio

#### Explanations

a. Serious imprecision: The 95% CIs around the absolute effect does not cross the null but includes potential small to large benefits using a population perspective.

b. Serious imprecision: The 95% CIs around the absolute effect crosses the null and includes potential meaningful harms and benefits using a population perspective.
