# Supplementary material for: Community-Based Child Food Interventions/Supplements for the Prevention of Wasting in Children Up to 5 Years at Risk of Wasting and Nutritional Oedema: A Systematic Review and Meta-Analysis
Source: Nutr Rev. 2025 Apr 24;83(8):1402–24. doi: 10.1093/nutrit/nuaf041 (PMC12241862; doi:10.1093/nutrit/nuaf041)
Supplement: nuaf041_Supplementary_Data [file nuaf041_supplementary_data.zip › nuaf041_Supplementary_Data/Supporting file 2.docx]

**Supporting file 2: Definition of Infants at risk of growth and development**

The key aim of 2013/24 WHO guideline process was to produce guidance on the identification and appropriate interventions for infants less than 6 months old who are not growing well, before they meet criteria for wasting and/or nutritional oedema.

A number of current WHO guidelines have nutritional/feeding recommendations and good practice statements for infants up to 6 weeks of age, however, there is a well-recognized gap in guidance between approximately 2 months of age to 6 months of age for infants at risk of poor growth and development more broadly, which includes but is not limited to severe wasting or nutritional oedema. Furthermore, most national nutrition guidelines and recommendations start when infants are at least 6 months of age. The Guideline Development Group (GDG) for the WHO guideline on prevention and management of wasting and nutritional oedema (acute malnutrition) was convened in September 2022 to determine an appropriate categorization of this broad population and potential identifiers of these infants, to whom recommendations and good practice statements in this guideline should apply.

The GDG agreed to call this population “infants at risk of poor growth and development.” The GDG proposed that, for the purpose of this guideline, infants at risk of poor growth and development should include infants less than 6 months of age in any of the following categories with any of the following criteria:

**Infants with poor growth based on sequential measures**

- No weight gain or weight loss from one measurement to the next; or
- Downward crossing of weight-for-age centile lines ; or
- Insufficient weight gain (velocity standards or grams/per specific time period)

Infants with poor anthropometry based on a single measure (if sequential measures not available)

- Weight-for-age z-score (WAZ) below -2 standard deviations (SD) of the WHO child growth standards median; or
- Weight-for-length z-score (WLZ) below -2 standard deviations (SD) of the WHO child growth standards median; or
- Nutritional oedema; or
- Mid-upper arm circumference (MUAC) less than 110 mm for infants between 6 weeks to less than 6 months of age

Infants with known risk factors for poor growth and development

- Neurodevelopmental concerns; or
- Infant feeding concerns; or
- Maternal risk (physical or mental health problem(s) affecting caring practices); or
- History of hospitalization

Infants at risk due to poor birth outcomes

- Preterm birth; or
- Low birth weight; or
- Small for gestational age

Note: Sequential measures are preferable to single measures, but other than this there is no hierarchy of these criteria; an infant can have any of these and be categorized as being at risk of poor growth and development.

**Reference:** World Health Organization. (2024). *WHO guideline on the prevention and management of wasting and nutritional oedema (acute malnutrition) in infants and children under 5 years*. World Health Organization. [Accessed from: <https://www.who.int/publications/i/item/9789240082830>]
