# Supplementary material for: Community-Based Child Food Interventions/Supplements for the Prevention of Wasting in Children Up to 5 Years at Risk of Wasting and Nutritional Oedema: A Systematic Review and Meta-Analysis
Source: Nutr Rev. 2025 Apr 24;83(8):1402–24. doi: 10.1093/nutrit/nuaf041 (PMC12241862; doi:10.1093/nutrit/nuaf041)
Supplement: nuaf041_Supplementary_Data [file nuaf041_supplementary_data.zip › nuaf041_Supplementary_Data/Supporting file 3.docx]

**Supporting file 2: Search strategy**

**Search strategy for articles published between 2021 and April 13, 2023.**

**MEDLINE**

1. exp infant/ or exp child, preschool/ or (infant* or toddler* or baby or babies or preschool or newborn* or neonate* or kindergarten or under-5* or "under 5*" or under-five or "under five" or kid or paediatr* or pediatr* or child*).mp.

AND

1. exp malnutrition/ or exp infant nutrition disorders/ or exp protein-energy malnutrition/ or exp wasting syndrome/ or (malnutrition or malnourish* or undernutrition or undernourish* or wasting or wasted).mp.

AND

1. exp diet/ or exp food/ or exp infant food/ or exp food, fortified/ or exp food, formulated/ or exp dietary supplements/ or exp food assistance/ or exp energy intake/ or exp dietary proteins/ or exp dietary fat/ or exp counseling/ or exp education/ or exp prenatal care/ or exp breast feeding/ or exp weaning/

OR

(diet* adj4 diversity or diet* adj4 quality or diet* adj4 variety or food* adj4 variety or nutri* adj4 diversity or nutri* adj4 adequacy or diet* adj4 pattern* or nutri* adj4 pattern* or "food assistance" or "food distribution" or "food aid" or "food program*" or supplement* or micronutrient* or "lipid-based nutrient supplement*" or “lipid nutrient supplement*” or blend* or nutributter or counselling or counseling or intervention* or strateg* or education* or "social protection" or "cash transfer*" or "cash incentive*" or voucher* or CCT or "income supplement*" or "social welfare" or "social polic*" or "food ration*" or "food basket*" or "breast feed*" or breastfeed* or "breast fed" or breastfed or EBF or "human milk" or “breast milk” or colostrum or "milk bank" or "feeding bank" or IYCF or "infant and young child feeding" or “young child feed*” or "infant feed*" or "complementary feed*" or "supplement* feed*" or "complementary food*" or "supplement* food*" or wean* or "infant food*" or "young child food*").mp.

**Ovid MEDLINE: Epub Ahead of Print, In-Process & Other Non-Indexed Citations, Ovid MEDLINE® Daily and Ovid MEDLINE® <1946-Present>**

1 exp infant/ or exp child, preschool/ or (infant* or toddler* or baby or babies or preschool* or newborn* or neonate* or kindergarten or under-5* or "under 5*" or under-five or "under five" or kid* or paediatr* or pediatr* or child*).mp.

2 exp malnutrition/ or exp infant nutrition disorders/ or exp protein-energy malnutrition/ or exp wasting syndrome/ or (malnutrition or malnourish* or undernutrition or undernourish* or wasting or wasted).mp.

3 exp diet/ or exp food/ or exp infant food/ or exp food, fortified/ or exp food, formulated/ or exp dietary supplements/ or exp food assistance/ or exp energy intake/ or exp dietary proteins/ or exp dietary fat/ or exp counseling/ or exp education/ or exp prenatal care/ or exp breast feeding/ or exp weaning/

4 ("dietary diversity" or "diet diversity" or "diet quality" or "dietary quality" or "dietary quality index" or "dietary variety" or "diet variety" or "dietary diversity score" or "food variety score" or "nutritional diversity" or "nutrient diversity" or "nutritional functional diversity" or "nutritional adequacy" or "nutrient adequacy" or "nutrition adequacy" or "dietary pattern*" or "diet pattern*" or "nutritional pattern*" or "nutrition pattern*" or "food assistance" or "food distribution" or "food aid" or "nutrition assistance" or "food program*" or supplement* or micronutrient* or "lipid-based nutrient supplement*" or "lipid nutrient supplement" or blend* or nutributter or counselling or counseling or intervention* or strateg* or education* or "social protection" or "cash transfer*" or "cash incentive*" or CCT or "income supplement*" or "social welfare" or "social polic*" or "food ration*" or "food basket*" or voucher* or "breast feed*" or breastfeed* or "breast fed" or breastfed or "human milk" or "breast milk" or colostrum or "milk bank" or "feeding bank" or IYCF or "infant and young child feeding" or "young child feed*" or "infant feed*" or "complementary feed*" or "supplement* feed*" or "complementary food*" or "supplement* food*" or wean* or "infant food*" or "young child food*").mp.

5 3 or 4

6 1 and 2 and 5

7 limit 6 to yr="2021 -Current"
