# Supplementary material for: Community-Based Child Food Interventions/Supplements for the Prevention of Wasting in Children Up to 5 Years at Risk of Wasting and Nutritional Oedema: A Systematic Review and Meta-Analysis
Source: Nutr Rev. 2025 Apr 24;83(8):1402–24. doi: 10.1093/nutrit/nuaf041 (PMC12241862; doi:10.1093/nutrit/nuaf041)

**Supporting file 3: Risk of Bias** **assessments of morbidity and mortality outcomes**

For RCTs:

D1 Randomisation process

D2 Deviations from the intended interventions

D3 Missing outcome data

D4 Measurement of the outcome

D5 Selection of the reported result

For cRCTs:

D1a Randomisation process

D1b Timing of identification or recruitment of participants

D2 Deviations from the intended interventions

D3 Missing outcome data

D4 Measurement of the outcome

D5 Selection of the reported result

**Fortified Blended Food – (infant/child)**

**Morbidity outcomes**


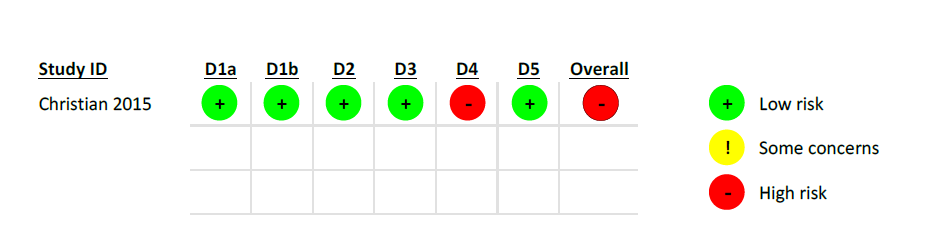

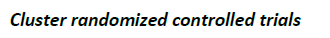


**Mortality outcomes**


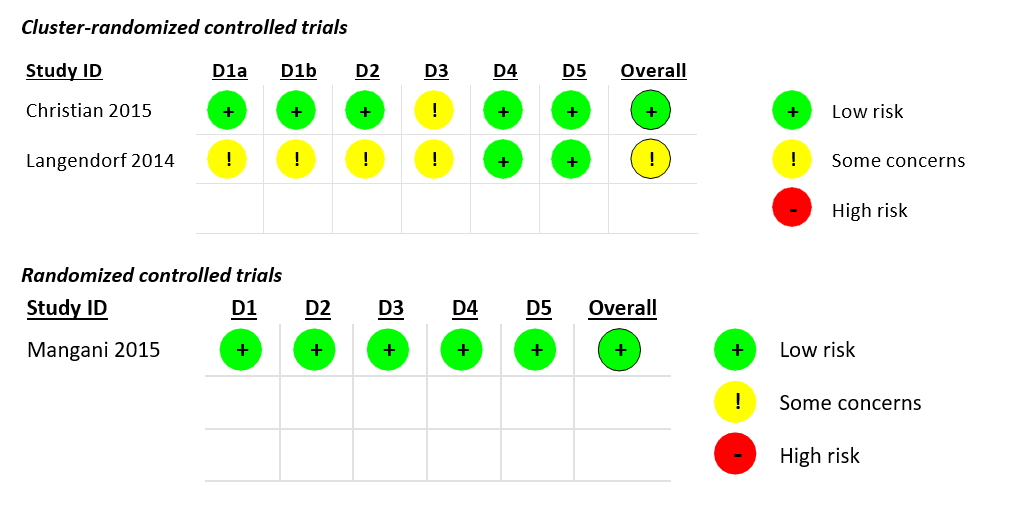

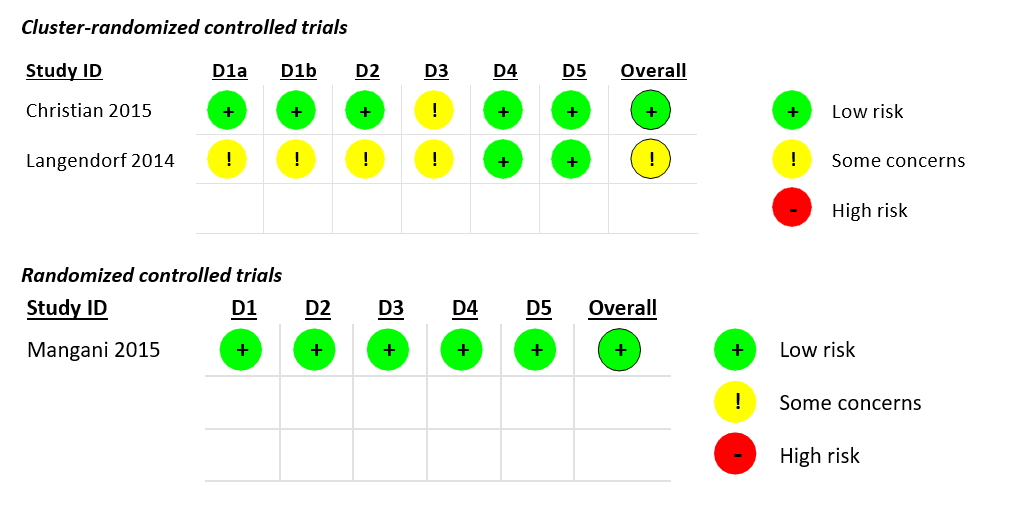


**LQ-LNS LQ and MQ-LNS - (infant/child)**

**Morbidity outcomes**


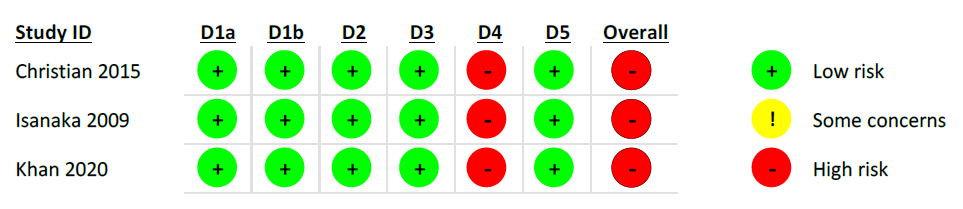


**Mortality outcomes**


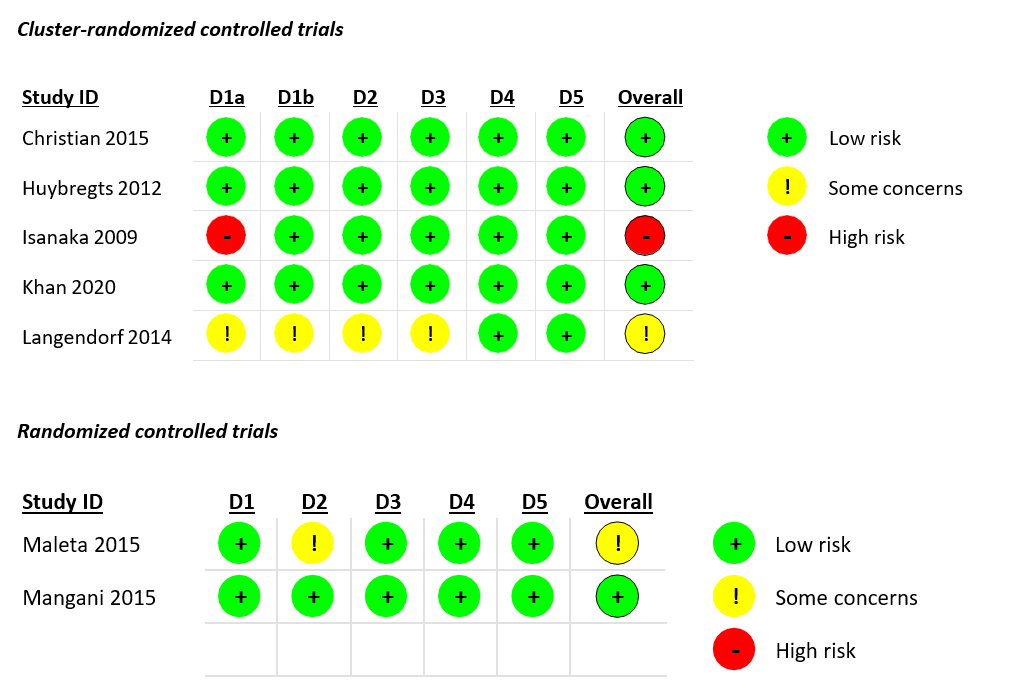

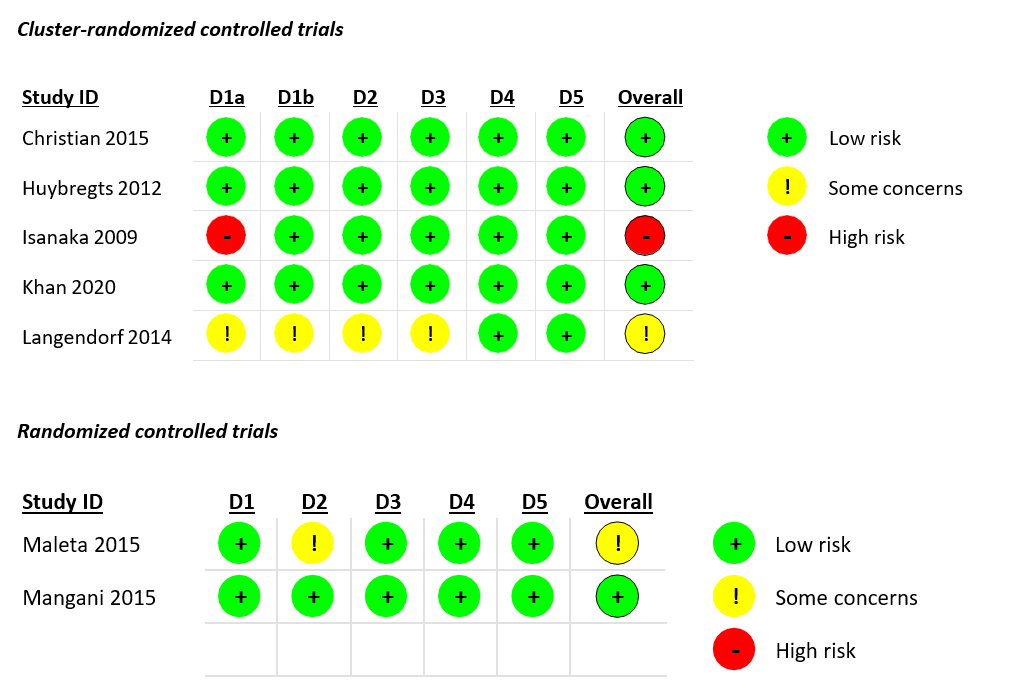


**SQ-LNS - (infant/child)**


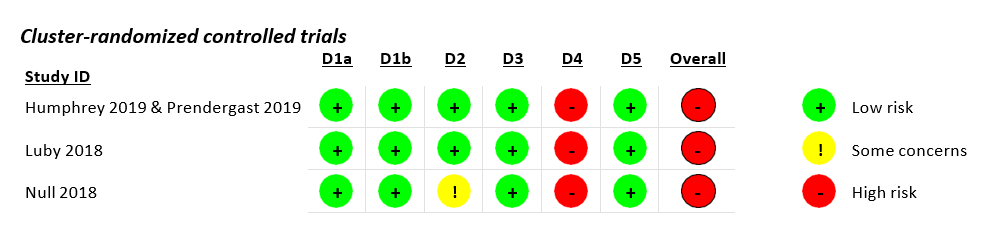
**Morbidity outcomes**


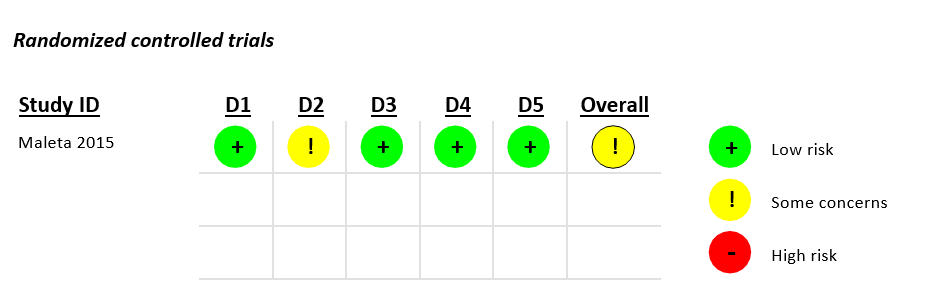

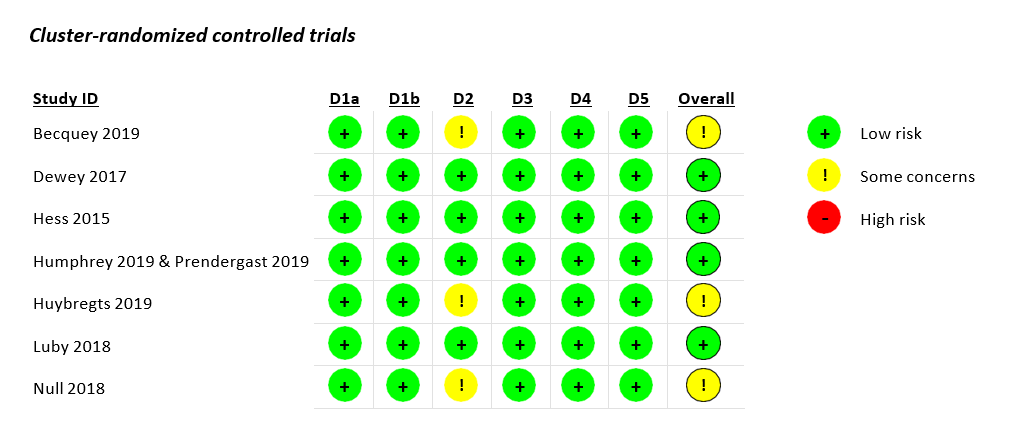
**Mortality outcomes**

**Micronutrient Powders - (infant/child)**


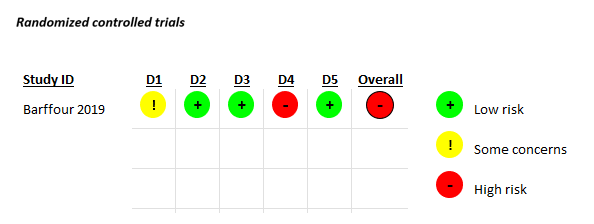

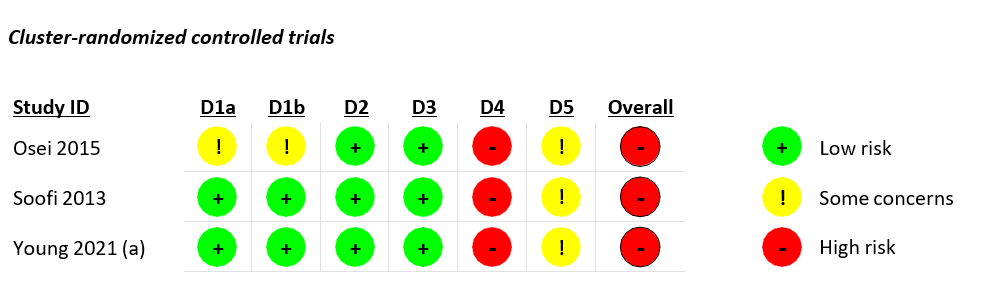
**Morbidity outcomes**


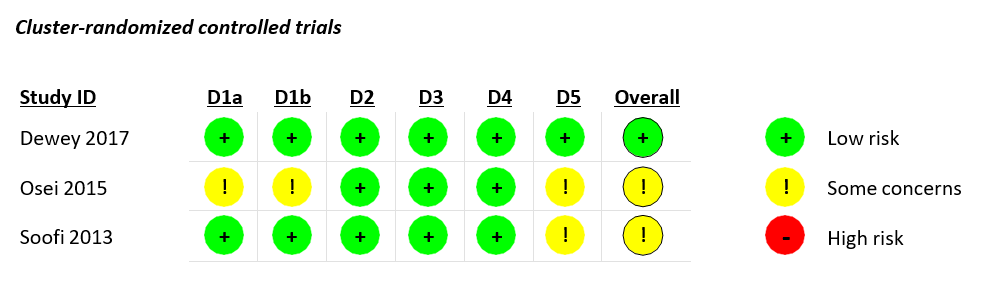
**Mortality outcomes**

**SQ-LNS - (maternal and infant/child)**


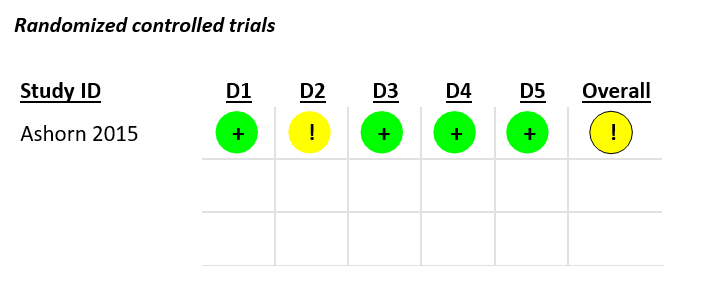
**Morbidity**

**Mortality**


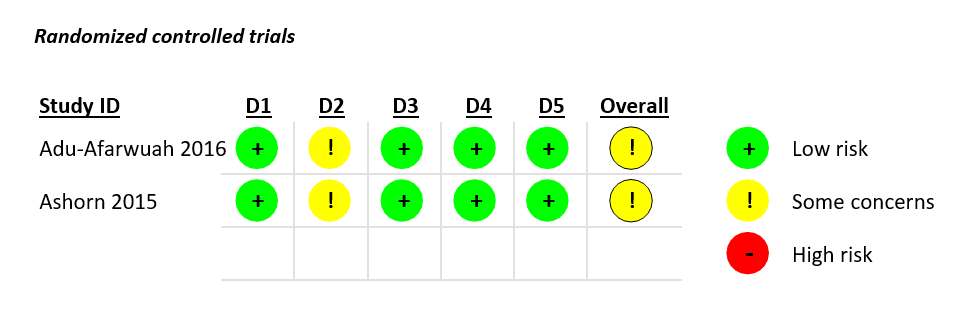

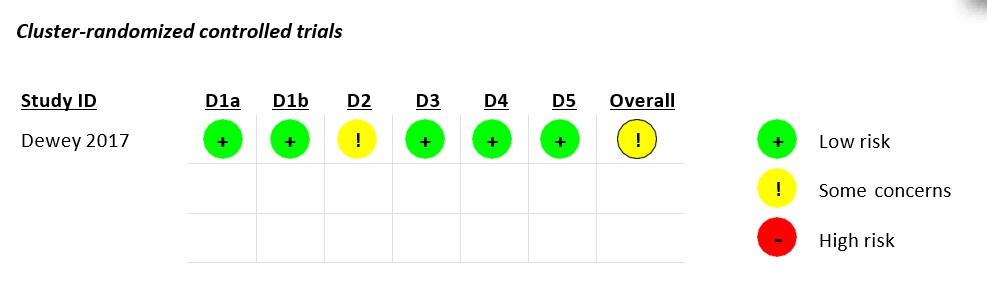

Supplement: nuaf041_Supplementary_Data [file nuaf041_supplementary_data.zip › nuaf041_Supplementary_Data/Supporting file 4.docx]
