# Supplementary material for: Community-Based Child Food Interventions/Supplements for the Prevention of Wasting in Children Up to 5 Years at Risk of Wasting and Nutritional Oedema: A Systematic Review and Meta-Analysis
Source: Nutr Rev. 2025 Apr 24;83(8):1402–24. doi: 10.1093/nutrit/nuaf041 (PMC12241862; doi:10.1093/nutrit/nuaf041)
Supplement: nuaf041_Supplementary_Data [file nuaf041_supplementary_data.zip › nuaf041_Supplementary_Data/Supporting file 5.docx]

**Intervention:** FBFs - infant/child supplementation

| **Certainty assessment** | | | | | | | **№ of patients** | | **Effect** | | **Certainty** | **Importance** |
| --- | --- | --- | --- | --- | --- | --- | --- | --- | --- | --- | --- | --- |
| **№ of studies** | **Study design** | **Risk of bias** | **Inconsistency** | **Indirectness** | **Imprecision** | **Other considerations** | **FBFs - infant/child - UPDATED WITHOUT LANGENDORF** | **control** | **Relative (95% CI)** | **Absolute (95% CI)** |  |  |
| **Prevalence of wasting** | | | | | | | | | | | | |
| 3 | randomised trials | not serious | not serious | not serious | very serious^a^ | none | 97/738 (13.1%) | 145/1005 (14.4%) | **RR 1.05** (0.83 to 1.33) | **7 more per 1,000** (from 25 fewer to 48 more) | ⨁⨁◯◯ Low | CRITICAL |
| **Prevalence of severe wasting** | | | | | | | | | | | | |
| 1 | randomised trials | not serious | not serious | not serious | very serious^b^ | none | 3/180 (1.7%) | 2/184 (1.1%) | **RR 1.53** (0.26 to 9.07) | **6 more per 1,000** (from 8 fewer to 88 more) | ⨁⨁◯◯ Low | CRITICAL |
| **Cumulative incidence of severe wasting** | | | | | | | | | | | | |
| 1 | randomised trials | not serious | not serious | not serious | very serious^c^ | none | 3/209 (1.4%) | 2/209 (1.0%) | **RR 1.50** (0.25 to 8.88) | **5 more per 1,000** (from 7 fewer to 75 more) | ⨁⨁◯◯ Low | CRITICAL |
| **Deterioration to severe wasting - not measured** | | | | | | | | | | | | |
| - | - | - | - | - | - | - | - | - | - | - | - | CRITICAL |
| **WHZ** | | | | | | | | | | | | |
| 4 | randomised trials | not serious | not serious | not serious | serious^d^ | none | 921 | 1190 | - | MD **0.02 higher** (0.02 lower to 0.05 higher) | ⨁⨁⨁◯ Moderate | IMPORTANT |
| **MUAC (cm)** | | | | | | | | | | | | |
| 2 | randomised trials | not serious | not serious | not serious | very serious^e^ | none | 572 | 920 | - | MD **0.07 higher** (0.05 lower to 0.19 higher) | ⨁⨁◯◯ Low | IMPORTANT |
| **WAZ** | | | | | | | | | | | | |
| 4 | randomised trials | not serious | not serious | not serious | very serious^e^ | none | 921 | 1190 | - | MD **0.07 higher** (0.04 lower to 0.18 higher) | ⨁⨁◯◯ Low | IMPORTANT |
| **Prevalence of underweight (WAZ <-2)** | | | | | | | | | | | | |
| 3 | randomised trials | not serious | not serious | not serious | very serious^a^ | none | 225/738 (30.5%) | 350/1005 (34.8%) | **RR 0.97** (0.71 to 1.32) | **10 fewer per 1,000** (from 101 fewer to 111 more) | ⨁⨁◯◯ Low | IMPORTANT |
| **Incidence of pneumonia** | | | | | | | | | | | | |
| 1 | randomised trials | serious^f^ | not serious | not serious | serious^g^ | none | 0/0 | 0/0 | **RR 0.89** (0.69 to 1.14) | **1 fewer per 1,000** (from 1 fewer to 1 fewer) | ⨁⨁◯◯ Low | IMPORTANT |
| **Incidence of diarrhea** | | | | | | | | | | | | |
| 1 | randomised trials | serious^f^ | not serious | not serious | serious^g^ | none | 0/0 | 0/0 | **RR 0.97** (0.83 to 1.13) | **1 fewer per 1,000** (from 1 fewer to 1 fewer) | ⨁⨁◯◯ Low | IMPORTANT |
| **Mortality** | | | | | | | | | | | | |
| 2 | randomised trials | not serious | not serious | not serious | serious^h^ | none | 13/785 (1.7%) | 17/1184 (1.4%) | **RR 0.97** (0.47 to 2.00) | **0 fewer per 1,000** (from 8 fewer to 14 more) | ⨁⨁⨁◯ Moderate | IMPORTANT |

**CI:** confidence interval; **MD:** mean difference; **RR:** risk ratio

#### Explanations

a. Very serious imprecision: The 95% CIs around the absolute effect crosses the null and includes moderate benefit and large harms using a population perspective.

b. Very serious imprecision: The 95% CIs around the absolute effect does cross the null and includes potentially small benefit and large harms using a population perspective.

c. Very serious imprecision: The 95% CIs around the absolute effect does cross the null and includes potentially small benefit and large harms using a population perspective. Relative effect is very wide with very few events.

d. Serious imprecision: The 95% CIs around the absolute effect crosses the null and includes potential trivial harms and meaningful benefits using a population perspective.

e. Very serious imprecision: The 95% CIs around the absolute effect crosses the null and includes potential trivial harms and meaningful benefits using a population perspective.

f. Serious risk of bias: One study with overall high risk of bias (Christian 2015).

g. Serious imprecision: The 95% CIs around the relative effect crosses the null and includes potential harms and benefits. Absolute effects not available.

h. Serious imprecision: The 95% CIs around the absolute effect crosses the null and includes potentially small benefits and harms using a population perspective.
