# Supplementary material for: Community-Based Child Food Interventions/Supplements for the Prevention of Wasting in Children Up to 5 Years at Risk of Wasting and Nutritional Oedema: A Systematic Review and Meta-Analysis
Source: Nutr Rev. 2025 Apr 24;83(8):1402–24. doi: 10.1093/nutrit/nuaf041 (PMC12241862; doi:10.1093/nutrit/nuaf041)
Supplement: nuaf041_Supplementary_Data [file nuaf041_supplementary_data.zip › nuaf041_Supplementary_Data/Supporting file 6.docx]

**Intervention:** LQ-LNS and MQ-LNS - infant/child supplementation

| **Certainty assessment** | | | | | | | **№ of patients** | | **Effect** | | **Certainty** | **Importance** |
| --- | --- | --- | --- | --- | --- | --- | --- | --- | --- | --- | --- | --- |
| **№ of studies** | **Study design** | **Risk of bias** | **Inconsistency** | **Indirectness** | **Imprecision** | **Other considerations** | **LQ-LNS and MQ-LNS - infant/child - UPDATED WITH SOOFI TRIAL AND WITHOUT LANGENDORF** | **control** | **Relative (95% CI)** | **Absolute (95% CI)** |  |  |
| **Prevalence of wasting** | | | | | | | | | | | | |
| 4 | randomised trials | not serious | not serious | not serious | serious^a^ | none | 327/2516 (13.0%) | 198/1452 (13.6%) | **RR 0.87** (0.74 to 1.03) | **18 fewer per 1,000** (from 35 fewer to 4 more) | ⨁⨁⨁◯ Moderate | CRITICAL |
| **Prevalence of severe wasting** | | | | | | | | | | | | |
| 1 | randomised trials | not serious | not serious | not serious | serious^b^ | none | 3/379 (0.8%) | 2/184 (1.1%) | **RR 0.73** (0.12 to 4.32) | **3 fewer per 1,000** (from 10 fewer to 36 more) | ⨁⨁⨁◯ Moderate | CRITICAL |
| **Incidence of wasting** | | | | | | | | | | | | |
| 2 | randomised trials | not serious | not serious | not serious | serious^c^ | none | 0/0 | 0/0 | **RR 0.74** (0.55 to 0.99)^d^ | **1 fewer per 1,000** (from 1 fewer to 1 fewer) | ⨁⨁⨁◯ Moderate | CRITICAL |
| **Cumulative incidence of wasting** | | | | | | | | | | | | |
| 3 | randomised trials | not serious | not serious^e^ | not serious | very serious^f^ | none | 269/1130 (23.8%) | 197/696 (28.3%) | **RR 0.77** (0.53 to 1.12) | **65 fewer per 1,000** (from 133 fewer to 34 more) | ⨁⨁◯◯ Low | CRITICAL |
| **Cumulative incidence of severe wasting** | | | | | | | | | | | | |
| 3 | randomised trials | not serious | not serious | not serious | serious^g^ | none | 25/1791 (1.4%) | 41/1389 (3.0%) | **RR 0.55** (0.32 to 0.92) | **13 fewer per 1,000** (from 20 fewer to 2 fewer) | ⨁⨁⨁◯ Moderate | CRITICAL |
| **Incidence of severe wasting -** | | | | | | | | | | | | |
| 1 | randomised trials | serious^h^ | not serious | not serious | not serious | none | 0/0 | 0/0 | **RR 0.45** (0.24 to 0.83)^d^ | **0 fewer per 1,000** (from 1 fewer to 0 fewer) | ⨁⨁⨁◯ Moderate | CRITICAL |
| **Deterioration to severe wasting - not measured** | | | | | | | | | | | | |
| - | - | - | - | - | - | - | - | - | - | - | - | CRITICAL |
| **WHZ** | | | | | | | | | | | | |
| 7 | randomised trials | not serious | not serious | not serious | serious^i^ | none | 4136 | 2821 | - | MD **0.03 higher** (0.04 lower to 0.11 higher) | ⨁⨁⨁◯ Moderate | IMPORTANT |
| **MUAC (cm)** | | | | | | | | | | | | |
| 4 | randomised trials | not serious | not serious | not serious | serious^i^ | none | 2618 | 1444 | - | MD **0.14 higher** (0.08 higher to 0.2 higher) | ⨁⨁⨁◯ Moderate | IMPORTANT |
| **WAZ** | | | | | | | | | | | | |
| 5 | randomised trials | not serious | not serious | not serious | serious^i^ | none | 3032 | 1871 | - | MD **0.03 higher** (0 to 0.06 higher) | ⨁⨁⨁◯ Moderate | IMPORTANT |
| **Prevalence of underweight (WAZ <-2)** | | | | | | | | | | | | |
| 4 | randomised trials | not serious | not serious | not serious | serious^j^ | none | 831/2879 (28.9%) | 456/1557 (29.3%) | **RR 0.91** (0.83 to 1.00) | **26 fewer per 1,000** (from 50 fewer to 0 fewer) | ⨁⨁⨁◯ Moderate | IMPORTANT |
| **Incidence of pneumonia or respiratory tract infection** | | | | | | | | | | | | |
| 3 | randomised trials | serious^k^ | not serious | not serious | serious^l^ | none | 0/0 | 0/0 | **RR 0.92** (0.80 to 1.07) | **1 fewer per 1,000** (from 1 fewer to 1 fewer) | ⨁⨁◯◯ Low | IMPORTANT |
| **Incidence of diarrhea** | | | | | | | | | | | | |
| 3 | randomised trials | serious^k^ | not serious | not serious | not serious | none | 0/0 | 0/0 | **RR 0.97** (0.90 to 1.04) | **1 fewer per 1,000** (from 1 fewer to 1 fewer) | ⨁⨁⨁◯ Moderate | IMPORTANT |
| **Mortality** | | | | | | | | | | | | |
| 7 | randomised trials | not serious | not serious | not serious | serious^m^ | none | 63/5223 (1.2%) | 52/2995 (1.7%) | **RR 0.60** (0.37 to 0.98) | **7 fewer per 1,000** (from 11 fewer to 0 fewer) | ⨁⨁⨁◯ Moderate | IMPORTANT |
|  | | | | | | | | | | | | |
|  | | | | | | | | | | | | |

**CI:** confidence interval; **MD:** mean difference; **RR:** risk ratio

#### Explanations

a. Serious imprecision: The 95% CIs around the absolute effect does cross the null threshold and includes potentially moderate benefits to trivial harms using a population perspective.

b. Serious imprecision: The 95% CIs around the absolute effect crosses the null and includes potential small benefit and moderate harms using a population perspective.

c. Serious imprecision: The 95% CIs around the relative effect is very close to the null and includes potential trivial and meaningful benefits. Absolute effects not available.

d. These data represent the incidence rate ratio.

e. Serious inconsistency: Not downgraded as this uncertainty is already considered in the single downgrade for imprecision (considering the random effects model) and does not warrant an additional downgrade.

f. Very serious imprecision: The 95% CIs around the absolute effect crosses the null threshold and includes potential meaningful harms and very large benefits using a population perspective.

g. Serious imprecision: The 95% CIs around the absolute effect does not cross the null threshold but includes potentially trivial to meaningful benefits using a population perspective.

h. Serious risk of bias: One study with high overall risk of bias (Isanaka 2009).

i. Serious imprecision: The 95% CIs around the absolute effect does not cross the null threshold but includes potentially trivial to meaningful benefits using a population perspective.

j. Serious imprecision: The 95% CIs around the absolute effect does not cross the null threshold but includes potentially trivial to meaningful benefits using a population perspective.

k. Serious risk of bias: All trials judged as overall high risk of bias.

l. Serious imprecision: The 95% CIs around the relative effect crosses the null and includes potential meaningful harms and benefits. Absolute effects not available.

m. Serious imprecision: The 95% CIs around the absolute effect does not cross the null threshold and includes trivial to small benefit.
