# Supplementary material for: Community-Based Child Food Interventions/Supplements for the Prevention of Wasting in Children Up to 5 Years at Risk of Wasting and Nutritional Oedema: A Systematic Review and Meta-Analysis
Source: Nutr Rev. 2025 Apr 24;83(8):1402–24. doi: 10.1093/nutrit/nuaf041 (PMC12241862; doi:10.1093/nutrit/nuaf041)
Supplement: nuaf041_Supplementary_Data [file nuaf041_supplementary_data.zip › nuaf041_Supplementary_Data/Supporting file 7.docx]

**Intervention:** SQ-LNS - infant/child supplementation

| **Certainty assessment** | | | | | | | **№ of patients** | | **Effect** | | **Certainty** | **Importance** |
| --- | --- | --- | --- | --- | --- | --- | --- | --- | --- | --- | --- | --- |
| **№ of studies** | **Study design** | **Risk of bias** | **Inconsistency** | **Indirectness** | **Imprecision** | **Other considerations** | **SQ-LNS - infant/child** | **control** | **Relative (95% CI)** | **Absolute (95% CI)** |  |  |
| **Prevalence of wasting** | | | | | | | | | | | | |
| 8 | randomised trials | not serious | not serious | not serious | serious^a^ | none | 565/6533 (8.6%) | 681/7810 (8.7%) | **RR 0.88** (0.79 to 0.98) | **10 fewer per 1,000** (from 18 fewer to 2 fewer) | ⨁⨁⨁◯ Moderate | CRITICAL |
| **Prevalence of moderate wasting** | | | | | | | | | | | | |
| 2 | randomised trials | not serious | not serious | not serious | serious^b^ | none | 210/1962 (10.7%) | 215/1978 (10.9%) | **RR 0.98** (0.82 to 1.18) | **2 fewer per 1,000** (from 20 fewer to 20 more) | ⨁⨁⨁◯ Moderate | CRITICAL |
| **Prevalence of severe wasting** | | | | | | | | | | | | |
| 2 | randomised trials | not serious | not serious^c^ | not serious | serious^d^ | none | 26/1450 (1.8%) | 34/1472 (2.3%) | **RR 0.78** (0.45 to 1.33) | **5 fewer per 1,000** (from 13 fewer to 8 more) | ⨁⨁⨁◯ Moderate | CRITICAL |
| **Incidence of wasting** | | | | | | | | | | | | |
| 2 | randomised trials | not serious | not serious | not serious | serious^e^ | none | 0/0 | 0/0 | **RR 0.76** (0.57 to 1.02)^f^ | **1 fewer per 1,000** (from 1 fewer to 1 fewer) | ⨁⨁⨁◯ Moderate | CRITICAL |
| **Cumulative incidence of wasting** | | | | | | | | | | | | |
| 1 | randomised trials | not serious | not serious | not serious | serious^g^ | none | 44/521 (8.4%) | 23/256 (9.0%) | **RR 0.94** (0.58 to 1.52) | **5 fewer per 1,000** (from 38 fewer to 47 more) | ⨁⨁⨁◯ Moderate | CRITICAL |
| **Cumulative incidence of severe wasting** | | | | | | | | | | | | |
| 1 | randomised trials | not serious | not serious | not serious | very serious^d^ | none | 11/528 (2.1%) | 3/262 (1.1%) | **RR 1.82** (0.51 to 6.47) | **9 more per 1,000** (from 6 fewer to 63 more) | ⨁⨁◯◯ Low | CRITICAL |
| **WHZ** | | | | | | | | | | | | |
| 9 | randomised trials | not serious | not serious | not serious | serious^h^ | none | 7015 | 8364 | - | MD **0.08 higher** (0.03 higher to 0.12 higher) | ⨁⨁⨁◯ Moderate | IMPORTANT |
| **MUAC (cm)** | | | | | | | | | | | | |
| 4 | randomised trials | not serious | not serious^c^ | not serious | serious^i^ | none | 3043 | 2856 | - | MD **0.13 higher** (0 to 0.26 higher) | ⨁⨁⨁◯ Moderate | IMPORTANT |
| **MUACZ** | | | | | | | | | | | | |
| 4 | randomised trials | not serious | not serious | not serious | serious^i^ | none | 1897 | 1899 | - | MD **0.06 higher** (0 to 0.11 higher) | ⨁⨁⨁◯ Moderate | IMPORTANT |
| **WAZ** | | | | | | | | | | | | |
| 7 | randomised trials | not serious | not serious | not serious | not serious | none | 4661 | 6036 | - | MD **0.14 higher** (0.09 higher to 0.19 higher) | ⨁⨁⨁⨁ High | IMPORTANT |
| **Prevalence of underweight (WAZ <-2)** | | | | | | | | | | | | |
| 7 | randomised trials | not serious | not serious | not serious | not serious | none | 919/4873 (18.9%) | 1278/6019 (21.2%) | **RR 0.84** (0.78 to 0.90) | **34 fewer per 1,000** (from 47 fewer to 21 fewer) | ⨁⨁⨁⨁ High | IMPORTANT |
| **Prevalence of diarrhea** | | | | | | | | | | | | |
| 5 | randomised trials | serious^j^ | not serious | not serious | serious^k^ | none | 583/6037 (9.7%) | 1060/8971 (11.8%) | **RR 0.94** (0.80 to 1.11) | **7 fewer per 1,000** (from 24 fewer to 13 more) | ⨁⨁◯◯ Low | IMPORTANT |
| **Prevalence of cough or respiratory infection** | | | | | | | | | | | | |
| 2 | randomised trials | serious^j^ | not serious | not serious | not serious^l^ | none | 5/877 (0.6%) | 5/845 (0.6%) | **RR 0.96** (0.28 to 3.31) | **0 fewer per 1,000** (from 4 fewer to 14 more) | ⨁⨁⨁◯ Moderate | IMPORTANT |
| **Prevalence of acute lower respiratory infection** | | | | | | | | | | | | |
| 1 | randomised trials | serious^m^ | not serious | not serious | very serious^n^ | none | 51/506 (10.1%) | 47/524 (9.0%) | **RR 1.12** (0.77 to 1.64) | **11 more per 1,000** (from 21 fewer to 57 more) | ⨁◯◯◯ Very low | IMPORTANT |
| **Prevalence of high fever** | | | | | | | | | | | | |
| 1 | randomised trials | serious^m^ | not serious | not serious | very serious^n^ | none | 95/506 (18.8%) | 95/525 (18.1%) | **RR 1.04** (0.80 to 1.34) | **7 more per 1,000** (from 36 fewer to 62 more) | ⨁◯◯◯ Very low | IMPORTANT |
| **Mortality** | | | | | | | | | | | | |
| 9 | randomised trials | not serious | not serious | not serious | not serious | none | 186/6824 (2.7%) | 321/8413 (3.8%) | **RR 0.76** (0.63 to 0.91) | **9 fewer per 1,000** (from 14 fewer to 3 fewer) | ⨁⨁⨁⨁ High | IMPORTANT |

**CI:** confidence interval; **MD:** mean difference; **RR:** risk ratio

#### Explanations

a. Serious imprecision: The 95% CIs around the absolute effect does not cross the null threshold but includes trivial to potential moderate benefits using a population perspective.

b. Serious imprecision: The 95% CIs around the absolute effect crosses the null and includes potential moderate benefit and harms using a population perspective.

c. Serious inconsistency: Not downgraded as this uncertainty is already considered in the single downgrade for imprecision (considering the random effects model) and does not warrant an additional downgrade.

d. Serious imprecision: The 95% CIs around the absolute effect crosses the null and includes potential moderate benefit and harms using a population perspective.

e. Serious imprecision: The 95% CIs around the relative effect crosses the null and includes potential meaningful harms and benefits. Absolute effects not available.

f. These data represent the incidence rate ratio.

g. Serious imprecision: The 95% CIs around the absolute effect crosses the null and includes potential meaningful benefit and harms using a population perspective.

h. Serious imprecision: The 95% CIs around the absolute effect does not cross the null but includes potential trivial and meaningful benefit using a population perspective.

i. Serious imprecision: The 95% CIs around the absolute effect does not cross the null but includes no effect and meaningful benefit using a population perspective.

j. Serious risk of bias: All studies judged as overall high risk of bias.

k. Serious imprecision: The 95% CIs around the absolute effect crosses the null and includes potential meaningful benefit and harms using a population perspective.

l. No imprecision: Not downgraded, low baseline risk (rare events), further changes in relative effects are unlikely to result in meaningful changes in absolute effects.

m. Serious risk of bias: The only study (Dewey 2017) judged as overall high risk of bias.

n. Very serious imprecision: The 95% CIs around the absolute effect crosses the null threshold and includes potentially moderate to large harm to moderate benefits using a population perspective.
