# Supplementary material for: Community-Based Child Food Interventions/Supplements for the Prevention of Wasting in Children Up to 5 Years at Risk of Wasting and Nutritional Oedema: A Systematic Review and Meta-Analysis
Source: Nutr Rev. 2025 Apr 24;83(8):1402–24. doi: 10.1093/nutrit/nuaf041 (PMC12241862; doi:10.1093/nutrit/nuaf041)
Supplement: nuaf041_Supplementary_Data [file nuaf041_supplementary_data.zip › nuaf041_Supplementary_Data/Supporting file 8.docx]

**Intervention:** MNP - infant/child supplementation

| **Certainty assessment** | | | | | | | **№ of patients** | | **Effect** | | **Certainty** | **Importance** |
| --- | --- | --- | --- | --- | --- | --- | --- | --- | --- | --- | --- | --- |
| **№ of studies** | **Study design** | **Risk of bias** | **Inconsistency** | **Indirectness** | **Imprecision** | **Other considerations** | **MNPs - infant/child - UPDATED WITH CORRECT YOUNG DATA** | **control** | **Relative (95% CI)** | **Absolute (95% CI)** |  |  |
| **Prevalence of wasting** | | | | | | | | | | | | |
| 6 | randomised trials | not serious | not serious | not serious | serious^a^ | none | 464/3746 (12.4%) | 459/3713 (12.4%) | **RR 1.01** (0.84 to 1.21) | **1 more per 1,000** (from 20 fewer to 26 more) | ⨁⨁⨁◯ Moderate | CRITICAL |
| **Deterioration to severe wasting - not measured** | | | | | | | | | | | | |
| - | - | - | - | - | - | - | - | - | - | - | - | CRITICAL |
| **WHZ** | | | | | | | | | | | | |
| 5 | randomised trials | not serious | not serious | not serious | serious^b^ | none | 3124 | 3089 | - | MD **0.02 higher** (0.04 lower to 0.07 higher) | ⨁⨁⨁◯ Moderate | IMPORTANT |
| **MUAC (cm)** | | | | | | | | | | | | |
| 2 | randomised trials | not serious | not serious | not serious | serious^b^ | none | 781 | 817 | - | MD **0**  (0.09 lower to 0.09 higher) | ⨁⨁⨁◯ Moderate | IMPORTANT |
| **MUACZ** | | | | | | | | | | | | |
| 1 | randomised trials | not serious | not serious | not serious | serious^c^ | none | 894 | 816 | - | MD **0.05 lower** (0.13 lower to 0.03 higher) | ⨁⨁⨁◯ Moderate | IMPORTANT |
| **WAZ** | | | | | | | | | | | | |
| 4 | randomised trials | not serious | not serious^d^ | not serious | serious^b^ | none | 2423 | 2350 | - | MD **0**  (0.12 lower to 0.12 higher) | ⨁⨁⨁◯ Moderate | IMPORTANT |
| **Prevalence of underweight (WAZ <-2)** | | | | | | | | | | | | |
| 6 | randomised trials | not serious | not serious | not serious | serious^b^ | none | 1192/3815 (31.2%) | 1174/3799 (30.9%) | **RR 1.01** (0.94 to 1.08) | **3 more per 1,000** (from 19 fewer to 25 more) | ⨁⨁⨁◯ Moderate | IMPORTANT |
| **Prevalence of diarrhea** | | | | | | | | | | | | |
| 3 | randomised trials | serious^e^ | serious^f^ | not serious | not serious | none | 157/1906 (8.2%) | 214/1859 (11.5%) | **RR 0.72** (0.60 to 0.88) | **32 fewer per 1,000** (from 46 fewer to 14 fewer) | ⨁⨁◯◯ Low | IMPORTANT |
| **Incidence of diarrhea** | | | | | | | | | | | | |
| 1 | randomised trials | serious^g^ | not serious | not serious | serious^h^ | none | 0/0 | 0/0 | **RR 1.12** (1.00 to 1.25)^j^ | **1 fewer per 1,000** (from 1 fewer to 1 fewer) | ⨁⨁◯◯ Low | IMPORTANT |
| **Prevalence of fever or high fever** | | | | | | | | | | | | |
| 3 | randomised trials | serious^e^ | not serious | not serious | serious^j^ | none | 536/1906 (28.1%) | 577/1859 (31.0%) | **RR 0.92** (0.83 to 1.01) | **25 fewer per 1,000** (from 53 fewer to 3 more) | ⨁⨁◯◯ Low | IMPORTANT |
| **Incidence of fever** | | | | | | | | | | | | |
| 1 | randomised trials | serious^g^ | not serious | not serious | serious^h^ | none | 0/0 | 0/0 | **RR 0.95** (0.73 to 1.23)^j^ | **1 fewer per 1,000** (from 1 fewer to 1 fewer) | ⨁⨁◯◯ Low | IMPORTANT |
| **Incidence of rapid breathing or chest indrawing** | | | | | | | | | | | | |
| 1 | randomised trials | serious^g^ | not serious | not serious | not serious | none | 0/0 | 0/0 | **RR 1.61** (1.32 to 1.96)^j^ | **2 fewer per 1,000** (from 2 fewer to 1 fewer) | ⨁⨁⨁◯ Moderate | IMPORTANT |
| **Prevalence of acute lower respiratory infection** | | | | | | | | | | | | |
| 1 | randomised trials | serious^k^ | not serious | not serious | very serious^l^ | none | 43/567 (7.6%) | 47/524 (9.0%) | **RR 0.85** (0.57 to 1.26) | **13 fewer per 1,000** (from 39 fewer to 23 more) | ⨁◯◯◯ Very low | IMPORTANT |
| **Mortality** | | | | | | | | | | | | |
| 3 | randomised trials | not serious | not serious^f^ | not serious | serious^m^ | none | 26/1851 (1.4%) | 19/1701 (1.1%) | **RR 1.04** (0.35 to 3.06) | **0 fewer per 1,000** (from 7 fewer to 23 more) | ⨁⨁⨁◯ Moderate | IMPORTANT |

**CI:** confidence interval; **MD:** mean difference; **RR:** risk ratio

#### Explanations

1. Serious imprecision: The 95% CIs around the absolute effect crosses the null and includes potential benefit and harms using a population perspective.
2. Serious imprecision: The 95% CIs around the absolute effect crosses the null and includes potentially meaningful harms and benefits using a population perspective.
3. Serious imprecision: The 95% CIs around the absolute effect crosses the null and includes trivial benefits and potentially meaningful harms using a population perspective.
4. Serious inconsistency: Not downgraded as this uncertainty is already considered in the single downgrade for imprecision (considering the random effects model) and does not warrant an additional downgrade.
5. Serious risk of bias: All studies judged as having overall high risk of bias.
6. Serious inconsistency: Meaningful heterogeneity in study effects but undetected statistically due to small study bias, events and sample size.
7. Serious risk of bias: The study was judged as having overall high risk of bias.
8. Serious imprecision: The 95% CIs around the relative effect crosses the null and includes potential meaningful harms and benefits. Absolute effects not available.
9. These data represent the incidence rate ratio.
10. Serious imprecision: The 95% CIs around the absolute effect does not cross the null but includes potential large to trivial benefit using a population perspective.
11. Serious risk of bias: The only study (Dewey 2017) was judged as having high overall risk of bias.
12. Very serious imprecision: The 95% CIs around the absolute effect crosses the null and includes potentially moderate to large harms and benefits using a population perspective.
13. Serious imprecision: The 95% CIs around the absolute effect does cross the null and includes potentially trivial benefit to meaningful harms using a population perspective.
