# Supplementary material for: Community-Based Child Food Interventions/Supplements for the Prevention of Wasting in Children Up to 5 Years at Risk of Wasting and Nutritional Oedema: A Systematic Review and Meta-Analysis
Source: Nutr Rev. 2025 Apr 24;83(8):1402–24. doi: 10.1093/nutrit/nuaf041 (PMC12241862; doi:10.1093/nutrit/nuaf041)
Supplement: nuaf041_Supplementary_Data [file nuaf041_supplementary_data.zip › nuaf041_Supplementary_Data/Supporting file 9.docx]

**Intervention:** SQ-LNS - maternal & infant/child supplementation

| **Certainty assessment** | | | | | | | **№ of patients** | | **Effect** | | **Certainty** | **Importance** |
| --- | --- | --- | --- | --- | --- | --- | --- | --- | --- | --- | --- | --- |
| **№ of studies** | **Study design** | **Risk of bias** | **Inconsistency** | **Indirectness** | **Imprecision** | **Other considerations** | **SQ-LNS - maternal & infant/child** | **control** | **Relative (95% CI)** | **Absolute (95% CI)** |  |  |
| **Prevalence of wasting** | | | | | | | | | | | | |
| 3 | randomised trials | not serious | not serious | not serious | serious^a^ | none | 144/1445 (10.0%) | 191/1950 (9.8%) | **RR 0.86** (0.70 to 1.06) | **14 fewer per 1,000** (from 29 fewer to 6 more) | ⨁⨁⨁◯ Moderate | CRITICAL |
| **Prevalence of severe wasting** | | | | | | | | | | | | |
| 1 | randomised trials | not serious | not serious | not serious | very serious^b^ | none | 3/214 (1.4%) | 3/442 (0.7%) | **RR 2.07** (0.42 to 10.15) | **7 more per 1,000** (from 4 fewer to 62 more) | ⨁⨁◯◯ Low | CRITICAL |
| **Deterioration to severe wasting - not measured** | | | | | | | | | | | | |
| - | - | - | - | - | - | - | - | - | - | - | - | CRITICAL |
| **WHZ** | | | | | | | | | | | | |
| 3 | randomised trials | not serious | not serious | not serious | serious^a^ | none | 1445 | 1950 | - | MD **0.05 higher** (0.03 lower to 0.14 higher) | ⨁⨁⨁◯ Moderate | IMPORTANT |
| **MUAC (cm)** | | | | | | | | | | | | |
| 2 | randomised trials | not serious | not serious | not serious | serious^c^ | none | 562 | 1138 | - | MD **0.01 higher** (0.18 lower to 0.21 higher) | ⨁⨁⨁◯ Moderate | IMPORTANT |
| **MUACZ** | | | | | | | | | | | | |
| 2 | randomised trials | not serious | not serious | not serious | serious^c^ | none | 1098 | 1258 | - | MD **0.02 higher** (0.05 lower to 0.09 higher) | ⨁⨁⨁◯ Moderate | IMPORTANT |
| **WAZ** | | | | | | | | | | | | |
| 3 | randomised trials | not serious | not serious^d^ | not serious | serious^e^ | none | 1445 | 1951 | - | MD **0.1 higher** (0.03 lower to 0.23 higher) | ⨁⨁⨁◯ Moderate | IMPORTANT |
| **Prevalence of underweight (WAZ <-2)** | | | | | | | | | | | | |
| 3 | randomised trials | not serious | not serious | not serious | serious^f^ | none | 378/1445 (26.2%) | 452/1951 (23.2%) | **RR 0.93** (0.83 to 1.05) | **16 fewer per 1,000** (from 39 fewer to 12 more) | ⨁⨁⨁◯ Moderate | IMPORTANT |
| **Incidence of diarrhea (requiring hospitalization)** | | | | | | | | | | | | |
| 1 | randomised trials | not serious | not serious | not serious | serious^c^ | none | 4/335 (1.2%) | 14/696 (2.0%) | **RR 0.59** (0.20 to 1.79) | **8 fewer per 1,000** (from 16 fewer to 16 more) | ⨁⨁⨁◯ Moderate | IMPORTANT |
| **Prevalence of diarrhea** | | | | | | | | | | | | |
| 1 | randomised trials | serious^g^ | not serious | not serious | serious^h^ | none | 25/586 (4.3%) | 14/525 (2.7%) | **RR 1.60** (0.84 to 3.04) | **16 more per 1,000** (from 4 fewer to 54 more) | ⨁⨁◯◯ Low | IMPORTANT |
| **Incidence of pneumonia (requiring hospitalization)** | | | | | | | | | | | | |
| 1 | randomised trials | not serious | not serious | not serious | very serious^i^ | none | 18/335 (5.4%) | 53/696 (7.6%) | **RR 0.71** (0.42 to 1.19) | **22 fewer per 1,000** (from 44 fewer to 14 more) | ⨁⨁◯◯ Low | IMPORTANT |
| **Prevalence of acute lower respiratory infection** | | | | | | | | | | | | |
| 1 | randomised trials | serious^g^ | not serious | not serious | very serious^j^ | none | 50/586 (8.5%) | 47/524 (9.0%) | **RR 0.95** (0.65 to 1.39) | **4 fewer per 1,000** (from 31 fewer to 35 more) | ⨁◯◯◯ Very low | IMPORTANT |
| **Prevalence of high fever** | | | | | | | | | | | | |
| 1 | randomised trials | serious^g^ | not serious | not serious | very serious^k^ | none | 88/586 (15.0%) | 95/525 (18.1%) | **RR 0.83** (0.64 to 1.08) | **31 fewer per 1,000** (from 65 fewer to 14 more) | ⨁◯◯◯ Very low | IMPORTANT |
| **Mortality** | | | | | | | | | | | | |
| 3 | randomised trials | not serious | serious^d^ | not serious | serious^l^ | none | 11/1545 (0.7%) | 31/2171 (1.4%) | **RR 0.62** (0.24 to 1.58) | **5 fewer per 1,000** (from 11 fewer to 8 more) | ⨁⨁◯◯ Low | IMPORTANT |

**CI:** confidence interval; **MD:** mean difference; **RR:** risk ratio

#### Explanations

a. Serious imprecision: The 95% CIs around the absolute effect crosses the null threshold and includes potentially trivial harms to meaningful benefit using a population perspective.

b. Very serious imprecision: The 95% CIs around the absolute effect crosses the null threshold and includes potentially trivial benefits to very large harm using a population perspective.

c. Serious imprecision: The 95% CIs around the absolute effect crosses the null threshold and includes meaningful benefits and harms using a population perspective.

d. Serious inconsistency: Not downgraded as this uncertainty is already considered in the double downgrade for imprecision (considering the random effects model) and does not warrant an additional downgrade.

e. Serious imprecision: The 95% CIs around the absolute effect crosses the null threshold and includes potentially trivial harms and meaningful benefits using a population perspective.

f. Serious imprecision: The 95% CIs around the absolute effect crosses the null threshold and includes meaningful benefits and trivial harms using a population perspective.

g. Serious risk of bias: The only study (Dewey 2017) judged as overall high risk of bias.

h. Serious imprecision: The 95% CIs around the absolute effect crosses the null threshold and includes potential trivial benefits to moderate to large harms using a population perspective.

i. Very serious imprecision: The 95% CIs around the absolute effect crosses the null threshold and includes potentially meaningful harm to moderate to large benefits using a population perspective.

j. Very serious imprecision: The 95% CIs around the absolute effect crosses the null threshold and includes potentially moderate to large benefits and harm using a population perspective.

k. Very serious imprecision: The 95% CIs around the absolute effect crosses the null threshold and includes potentially large benefits to small harms using a population perspective.

l. Serious imprecision: The 95% CIs around the absolute effect crosses the null threshold and includes potentially meaningful benefits to trivial harm using a population perspective.
